# Jahn–Teller effects in Au_25_(SR)_18_†
†Electronic supplementary information (ESI) available: The supplemental information contains additional details about the syntheses and characterization of these compounds. It includes SQUID magnetometry, electrochemical methods, X-ray crystallography information, DFT methods and analysis and crystallographic data. CCDC 1055143 and 1055144. For ESI and crystallographic data in CIF or other electronic format see DOI: 10.1039/c5sc02134k


**DOI:** 10.1039/c5sc02134k

**Published:** 2015-11-24

**Authors:** Marcus A. Tofanelli, Kirsi Salorinne, Thomas W. Ni, Sami Malola, Brian Newell, Billy Phillips, Hannu Häkkinen, Christopher J. Ackerson

**Affiliations:** a Department of Chemistry , Colorado State University , Fort Collins , Colorado 80523 , USA . Email: ackerson@colostate.edu; b Departments of Chemistry and Physics , Nanoscience Center , University of Jyväskylä , FI-40014 Jyväskylä , Finland

## Abstract

The relationship between oxidation state, structure, and magnetism in many molecules is well described by first-order Jahn–Teller distortions. This relationship is not yet well defined for ligated nanoclusters and nanoparticles, especially the nano-technologically relevant gold-thiolate protected metal clusters. Here we interrogate the relationships between structure, magnetism, and oxidation state for the three stable oxidation states, –1, 0 and +1 of the thiolate protected nanocluster Au_25_(SR)_18_. We present the single crystal X-ray structures of the previously undetermined charge state Au_25_(SR)_18_^+1^, as well as a higher quality single crystal structure of the neutral compound Au_25_(SR)_18_^0^. Structural data combined with SQUID magnetometry and DFT theory enable a complete description of the optical and magnetic properties of Au_25_(SR)_18_ in the three oxidation states. In aggregate the data suggests a first-order Jahn–Teller distortion in this compound. The high quality single crystal X-ray structure enables an analysis of the ligand–ligand and ligand–cluster packing interactions that underlie single-crystal formation in thiolate protected metal clusters.

## Abstract 2

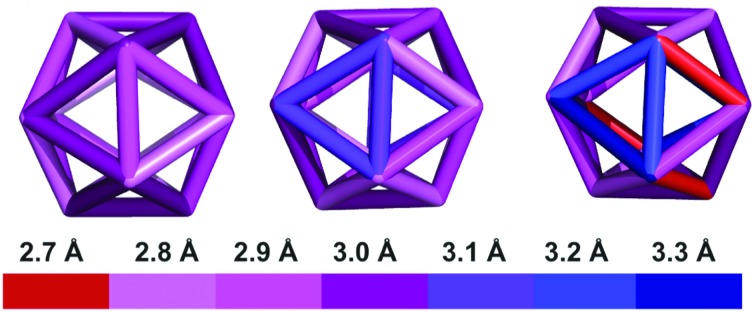
Jahn–Teller distortions are observed in Au_25_(SR)_18_ by single crystal X-ray crystallography. SQUID magnetometry, DFT theory, and linear optical spectroscopy corroborate the finding.

## Introduction

The Jahn–Teller theorem establishes that molecular orbitals must be symmetrically occupied by electrons in order for them to be energetically degenerate.^1^ Unequal occupation of orbitals leads to breaking of the energetic degeneracy of the orbitals, with concomitant distortions to the symmetry of the molecule, coupled to simultaneous changes in optical and magnetic properties. Jahn–Teller effects are described experimentally for low-nuclearity metal clusters,^2^ carbon clusters such as fullerenes,^3^ clusters in extended solids,^4^ Zintl phases,^5^ and theoretically for larger nanoclusters.^6^–^8^

For nanocluster compounds (here we define a nanocluster as a metal cluster with one or more metal atoms that is neighbored only by other metal atoms) the role of the Jahn–Teller effect is unclear. In this work, we investigate the structural and magnetic properties of Au_25_(SR)_18_ in 3 charge states. Of the compounds comprising the Au_*x*_(SR)_*y*_ monolayer protected cluster magic number series,^9^ the Au_25_(SR)_18_ nanocluster^10^,^11^ is the best understood, both experimentally and theoretically. The compound was initially isolated by Whetten,^10^ with the Au_25_(SR)_18_ formulation made subsequently by Tsukuda.^12^ The single-crystal X-ray structure^13^,^14^ combined with reliable syntheses^15^,^16^ preceded the emergence of this compound as a singular subject for understanding the physical and inorganic chemistry of broadly studied and applied^17^,^18^ thiolate protected gold nanoclusters.

Theoretical studies conclude that the frontier orbitals of Au_25_(SR)_18_ and many other Au_*x*_(SR)_*y*_ compounds as large as Au_102_(SR)_44_ are well predicted by a spherical superatom model.^9^,^19^ In this model, Au_25_(SR)_18_^–1^ is an 8e^–^ system, corresponding to a noble gas-like 1S^2^1P^6^ superatom electron configuration. The superatom electron configuration of Au_25_(SR)_18_ can be modified through now well established electrochemical methods which allows for stable preparations of Au_25_(SR)_18_ in –1, 0 and +1 oxidation states, corresponding to 1S^2^1P^6^, 1S^2^1P^5^, and 1S^2^1P^4^ superatom electron configurations, respectively. Several properties including magnetism, optical absorption, catalytic reactivity and stability can be rationalized in terms of superatom electron configuration.^14^,^20^,^21^ Of these reports, magnetic studies may give insight into whether Au_25_(SR)_18_ is subject to Jahn–Teller effects.

If Jahn–Teller effects do not apply to Au_25_(SR)_18_, then Hund's rule predicts that the –1, 0, and +1 charge states should be diamagnetic, *S* = 1/2 paramagnetic and *S* = 1 paramagnetic, respectively. However, if the cluster has morphological flexibility and can change shape with changing charge, then the superatomic orbitals may lose their degeneracy with changing charge states and the –1, 0 and +1 charge states would become diamagnetic, *S* = 1/2 paramagnetic, and diamagnetic, respectively. The magnetic properties of thiolate protected gold nanoparticles, however, are controversial, with inconsistent reports of magnetic properties made for apparently similar preparations.^22^ Indeed, even for the remarkably well defined cluster Au_25_(SR)_18_ there are conflicting reports of magnetism. Of three prior reports interrogating Au_25_(SR)_18_ magnetism by EPR or NMR spectroscopy, all reports found that the –1 and 0 oxidation states are diamagnetic and *S* = 1/2 paramagnetic, consistent with superatom theory for the cluster. There are conflicting reports, however, regarding the nature of the +1 cluster, with two studies concluding diamagnetism and one study concluding paramagnetism.^21^,^23^–^25^

Here we present a comprehensive study on the structures, magnetic properties, and optical properties of Au_25_(PET)_18_ in its three stable charge states. Notably we present the first crystal structure of {[Au_25_(PET)_18_^+1^][PF_6_^–1^]}, as well as a notably higher resolution crystal structure of Au_25_(PET)_18_^0^ relative to a previous report.^26^ These structures show the same general atomic connectivity as observed in previous structures, with a 13 atom icosahedral core protected by 6 SR–Au–SR–Au–SR “semiring” units. The formal symmetry of the entire molecule, including the approximately icosahedral core, is *T*_h_.^27^ In addition, we make the first SQUID magnetometry study of all three charge states, and also present linear absorption spectra from redissolved crystals of each charge state, notably improving upon the previous spectroelectrochemistry of this compound. We observe geometric distortions away from idealized symmetry in the inorganic core, and these distortions increased with decreasing superatomic valence from 1S^2^1P^6^ to 1S^2^1P^4^. The evolution of structure, magnetism and optical properties with oxidation state can be understood in terms of Jahn–Teller effects.

## Experimental

Au_25_(PET)_18_^–1^ was synthesized by minor modification of a previously reported protocol.^15^ 1.00 g (2.54 mmol) of HAuCl_4_·3H_2_O and 1.56 g (2.85 mmol) of tetraoctylammonium bromide (TOAB) were dissolved in 70 ml of THF. This solution stirred for 15 min turning color from yellow to orange. 1.8 ml (13.4 mmol) of 2-phenylethanethiol (PET) was added. The reaction mixture stirred until turning clear, approximately 3 hours. A freshly prepared aqueous solution containing 965 mg (25.5 mmol) NaBH_4_ and 24 ml of water at 0 °C was rapidly added under vigorous stirring. Stirring continued for 2 days, in a covered (but not sealed) flask. The reaction mixture was dried, giving an oily product. The crude product was sonicated in methanol resulting in precipitation of the Au_25_(SR)_18_^–1^. The precipitate was collected by centrifugation and washed four times with methanol. Crystals of Au_25_(PET)_18_^–1^ were formed by dissolving Au_25_(PET)_18_^–1^ and TOAB in toluene and adding ethanol just until a precipitate forms and is collected by centrifugation. The precipitate is examined for the optical signature of Au_25_(SR)_18_^–1^. Additional fractions of ethanol are added until the precipitate contains Au_25_(PET)_18_^–1^. Au_25_(PET)_18_^–1^ can be oxidized to the Au_25_(SR)_18_^0^ by shaking in the presence of silica gel. Crystals of Au_25_(PET)_18_^0^ were formed in the same way as Au_25_(PET)_18_^–1^, except TOAB was not added to the solution.

Au_25_(PET)_18_^+1^ prepared by bulk electrolysis from crystallized Au_25_(PET)_18_^–1^. Au_25_(PET)_18_^–1^ was dissolved in 0.1 M tetrabutylammonium hexafluorophosphate (TBAPF_6_) in dichloromethane (DCM). Bulk electrolysis was preformed at constant potential in a three-compartment cell at 300 mV *vs.* SCE. Immediately following electrolysis, the solution was taken for crystallization, as the lifetime of Au_25_(SR)_18_^+1^ is limited. Ethanol was added to the DCM solution used in bulk electrolysis until a precipitate formed that was collected by centrifugation. Dissolution in DCM followed by ethanol precipitation was repeated until Au_25_(PET)_18_^+1^ was isolated as judged by UV/Vis. The solution appears green. Au_25_(PET)_18_^+1^ can be stored as a dry precipitate at –20 °C. Crystals of –1/0/+1 oxidation states were determined by single crystal X-ray methods.

We performed density functional theory (DFT) calculations using the GPAW package that implements projector augmented-wave (PAW) method in a real-space grid.^25^ Electronic structure, charge distribution, magnetic states and optical absorption of the clusters in all charge states were analyzed. Crystal structure coordinates including the full ligand layer were used as such without structural relaxation to the theoretical minimum. The atomic charges were analyzed using the Bader decomposition method^26^ and the optical absorption spectra were calculated from the linear response time dependent DFT as implemented in GPAW.^27^ The PBE exchange–correlation functional was used both for the ground-state and optical absorption calculation. The PAW setups for gold include scalar-relativistic corrections.

## Results and discussion

### Symmetry analysis

**Fig. 1:**
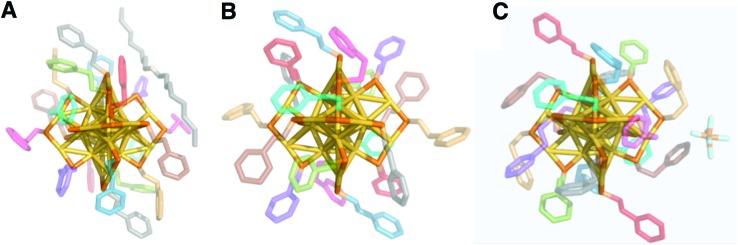
The crystal structures of Au_25_(PET)_18_ in the –1 (A), 0 (B), and +1 (C) charge states are shown above. Gold is in yellow, and sulfur is in orange. Crystallographically independent PET ligands are shown in unique color (see Table 1).

We report the crystal structure of Au_25_(PET)_18_^+1^ and an improved Au_25_(PET)_18_^0^ crystal structure.^24^ Each structure shows the same general atomic connectivity as observed previously,^13^,^14^,^26^,^28^ with each cluster structure containing a 13 gold atom filled icosahedral core surrounded by 6 SR–Au–SR–Au–SR “semi-rings”. A comparison of the structures of the crystallographically resolved charge states of Au_25_(PET)_18_^–1/0/+1^ (Fig. 1) reveals that the symmetry of the set of structures becomes less ideal similar to idealized polyhedral components as charge state increases.

**Table 1:** Geometric parameters and selected intra- and inter-cluster interactions of the PET ligands of the Au_25_(PET)_18_^+1^ crystal structure

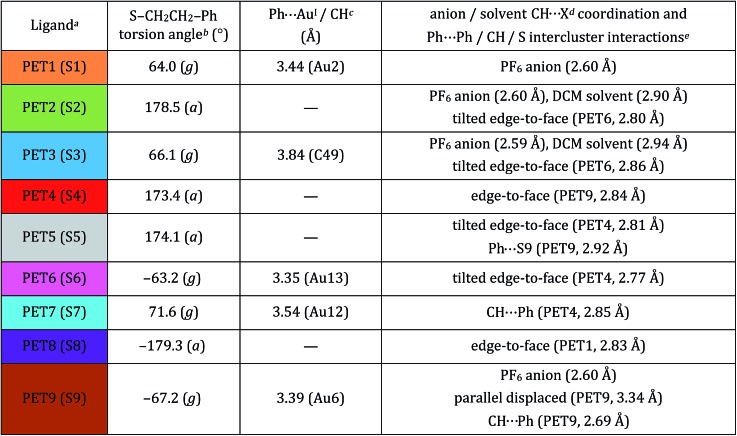

^*a*^Color code of the crystallographically independent PET ligand.

^*b*^ *g* = *gauche* and *a* = *anti*.

^*c*^Ligand intracluster interactions.

^*d*^X = halide (F or Cl).

^*e*^Average distance reported.

**Fig. 2:**
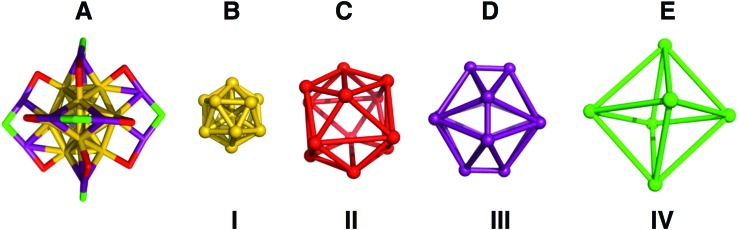
(A) Structures of the inorganic core and semirings of Au_25_(PET)_18_, with each color highlighting a different symmetry for sulfur or gold. In (B–E) the shape in which each unique atom makes is displayed, (B) is a gold icosahedron, (C) a distorted sulfur icosahedron, (D) a dodecahedron missing 8 vertices that form the vertices of an inscribed cube, each vertex shown corresponds to an Au(i) atom and (E) is a sulfur octahedron.

We quantified the distortions from ideal symmetry in two ways: first, by analysis of bond lengths, angles, and dihedral angles; second, by continuous symmetry measure (CSM)^29^,^30^ as implemented in SHAPE v2.1. CSM quantifies the deviation of a shape from its ideal counterpart by calculating the sum of squares of displacement from the ideal geometry. To implement CSM analysis, we developed a ‘shell-by-shell’ description of the geometric relationships of the atoms in Au_25_(SR)_18_ as shown in Fig. 2.

In the shell-by-shell description, Au_25_(PET)_18_ is composed of 4 shells of symmetrically related atoms (Fig. 2). The innermost shell (**I**, Fig. 2B) is a filled Au_12_ icosahedron. The next most outer shell (**II**, Fig. 2C) is comprised of 12 sulfur atoms that form the vertices of an icosahedron. The next most outer shell (**III**, Fig. 2D) is comprised of the 12 Au(i) atoms of Au_25_(SR)_18_ forming the vertices of an irregular polyhedron. This polyhedron can be considered as a dodecahedron missing 8 vertices. The missing 8 vertices inscribe a cube within the dodecahedron. The outermost shell (**IV**, Fig. 2E) is comprised of 6 sulfur atoms that form the vertices of an octahedron. The atoms in shell **I** are both chemically bonded and geometrically related as vertices of an icosahedron. In shells **II–IV** the atoms within each shells are related only by geometry and are not chemically bonded. Fig. 2A shows how these geometric shells are related in the context of chemical bonding in the structure.

The geometric relationships of the shells to each other is as follows: the S_12_ icosahedron of shell **II** caps each of the vertices of shell **I**. The 12 Au(i) atoms of shell **III**, in addition to being chemically bonded to **II** and **IV**, also face-cap 12 of the 20 faces of shell **I**. The S_12_ geometric icosahedron of shell **II** is distorted from the ideal shape in a manner consistent with its chemical bonding to shell **III**.

**Fig. 3:**
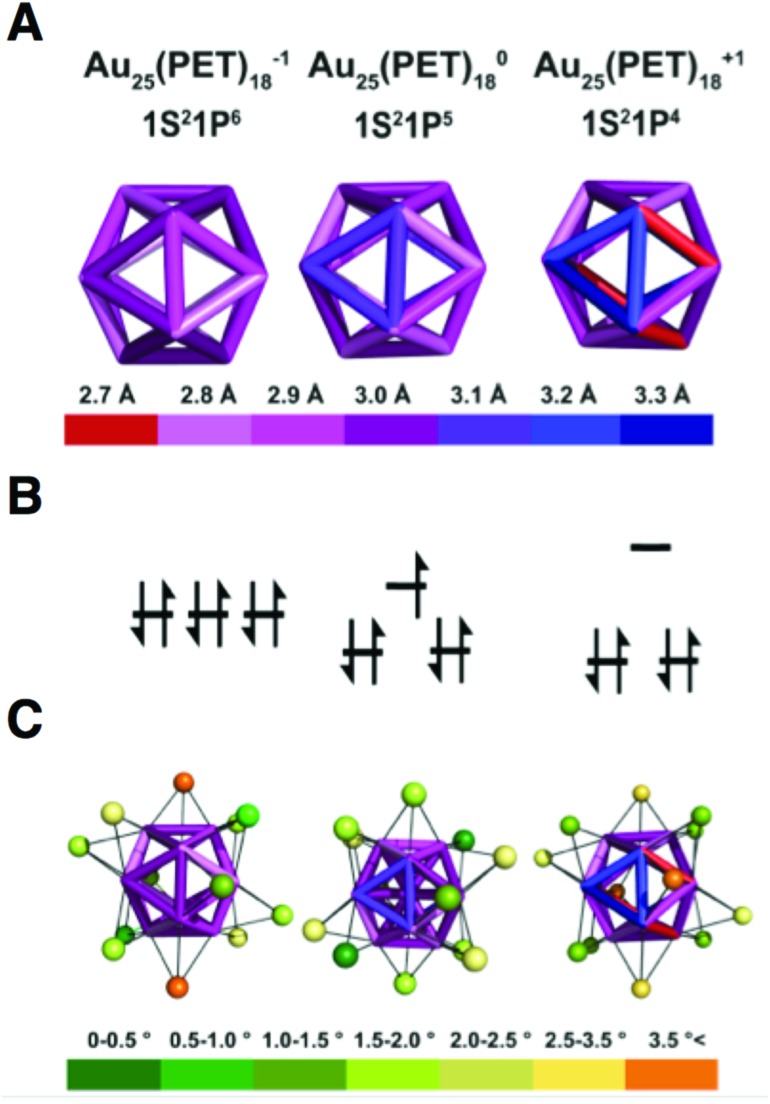
(A) A heat map of the edge bond length of the Au_12_ cores of Au_25_(SR)_18_^–1/0/+1^. (B) An energy level diagram of the P orbitals. (C) A heat map of the distortion away from face capping the Au(i) as judged by angles to the core.

CSM^30^ reveals shell **I** to be a nearly perfect icosahedron for Au_25_(PET)_18_^–1^. Increasing the oxidation state of Au_25_(PET)_18_ from the 8e^–^ superatom anion to 7e^–^ neutral and 6e^–^ cationic oxidation states results in distortion of shell **I**. CSM values for shell **I** relative to an ideal icosahedron are 0.067, 0.201 and 0.524 for the –1, 0 and +1 oxidation states, respectively. Bonds lengths for shell **I**, which in an ideal icosahedron are identical, vary over a range of 0.3 Å, 0.4 Å and 0.7 Å for Au_25_(PET)_18_^–1^, Au_25_(PET)_18_^0^, and Au_25_(PET)_18_^+1^, respectively. The variation in bond lengths is shown in a quantitative heat map of the icosahedral cores of each charge state in Fig. 3A. A summary of the bond lengths is given in ESI Table S2.†

The geometric distortions from **I** are also observed in shell **II**. The CSM values for shell **II** are 3.407, 3.879, and 4.45, for –1, to 0, and +1, respectively. For shell **III** the CSM values are 0.108, 0.092, and 0.155, for –1, 0 and +1, respectively. The CSM values for shell **IV** are 0.138, 0.109, and 0.106, for –1, 0, and +1.

The outer-most shell **IV** is apparently least affected by charge state, as it is almost ideally octahedral for 0 and +1, while –1 shows the largest deviation from this symmetry. We attribute the deviation from ideal symmetry in shell **IV** for the anion to the packing of the tetraoctyl ammonium cation in the crystal lattice. In the case of Au_25_(PET)_18_^+1^, the lattice position of the PF_6_^–^ ion does not cause deviation from ideal symmetry in shell **IV**.

To fully describe the changes that occur to the semi-rings (**II–IV**), we elaborate how each shell distorts with respect to shell **I**. The symmetry of the inorganic core (shells **I–IV**) is approximately of the point group *D*_2h_.^19^ This approximation assumes the semirings on opposite sides of the cluster are coplanar, with the other four semirings lying orthogonal to the plane defined by coplanar semirings. In all structures of Au_25_(SR)_18_, there is some deviation from this idealized description. The amount of symmetry lowering, on average, increases with increasing oxidation state. As Au_25_(PET)_18_ becomes more oxidized the gold atoms in shell **III** shift toward the edges of shell **I** in order to stabilize the longer/weaker bonds that arise from distortion of the icosahedron. As shown in ESI Table S4† the average degree that the atoms in shell **III** deviate away from the face are 1.91°, 2.06°, and 2.63° for –1, 0, and +1 charge states, respectively. As a consequence of the distortion of the underlying structure, atoms in shells **II** and **IV** bend out of the plane defined by the *D*_2h_ point group.

**Fig. 4:**
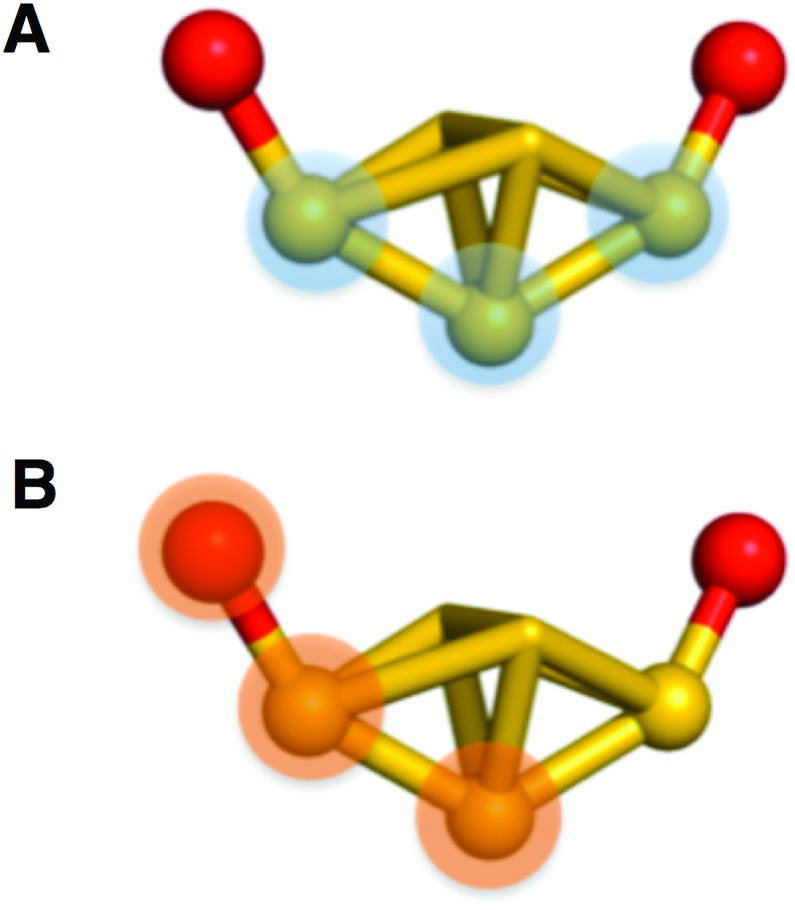
Depicted above is the two planes used to measure the dihedral angles of the semi-rings. (A) shows the plane defined by the core and (B) shows the plane defined by the semi-ring. The plane on (B) is changed to incorporate the appropriate atom in the semi-ring and is measured on both sides.

Measurement of the dihedral angles shown in Fig. 4 allows quantification of deviation from the ideal point group. One plane is contained within shell **I** and is defined as the central atom of the icosahedron and the two vertex gold atoms anchoring each side of a semiring (Fig. 4A). The second plane is defined by the atom of the semi-ring, the gold atom of shell **I** to which the semi-ring is anchored, and the central atom of the cluster (Fig. 4B).

Measured for all semi-rings, the average dihedral angles are 7.3°, 8.6°, and 12.8°, for –1, 0, and +1, respectively. Individual values are tabulated in Table S1.†

The deviations from ideal symmetry of **IV** identified by CSM are presently described independently of chemical bonding to other shells. Chemical bonding requirements in the cluster clearly influence the sulfur atoms of shell **IV**. This is most obvious in the dihedral angles of the atoms in the semi-rings. The sulfur atoms in **IV** have the largest average dihedral angle for the +1 (5.1°), followed by –1 (3.9°), and finally 0 (0.8°) oxidation states. Thus, the coordinates of atoms in shell **IV** appear to be influenced by a combination of counterion, solvent, and underlying inorganic structure.

We previously reported that the thermal stability of Au_25_(PET)_18_ depends on the superatomic electron configuration,^20^ with lower stabilities associated with departure from noble-gas like superatom electron configuration. This work suggests that the changes in cluster geometry that arise as charge state may be tied to the thermal stability we previously observed. For instance, we observe that the longest (weakest) bond in the icosahedral core is 3 Å, 3.1 Å, and 3.3 Å for –1, 0, and +1, respectively. These effects are also seen in shells **II** and **III**.

### Optical/electronic properties of Au_25_(PET)_18_^–1/0/+1^

The absorption spectra of Au_25_(PET)_18_ evolves notably across each charge state, suggesting changes in the underlying electronic structure after oxidation or reduction of Au_25_(SR)_18_. The absorption peak around 680 nm (1.81 eV) is attributed to the transition from the 1P to 1D_e_ and the peak between 450–470 nm (2.76–2.58 eV) has been attributed to the transition of the 1P to 1D_t_.^19^ The transition at 380–400 nm (3.15–3.08 eV) is attributed to energy states arising from semi-ring structure into the 1D_e_ orbital. The energy transition from the 1P to 1D_e_ for the –1, 0, and +1 are 1.78 eV, 1.81 eV, and 1.88 eV, respectively. For the 1P to 1D_t_ energy gaps of 2.76 eV, 2.68 eV, and 2.58 eV are observed for the –1, 0 and +1, respectively. Finally the energy gap for ligand band to superatomic D orbital transition is 3.08 eV for –1 and 0, and 3.15 eV for +1. These values are summarized in ESI Table S7.†

**Fig. 5:**
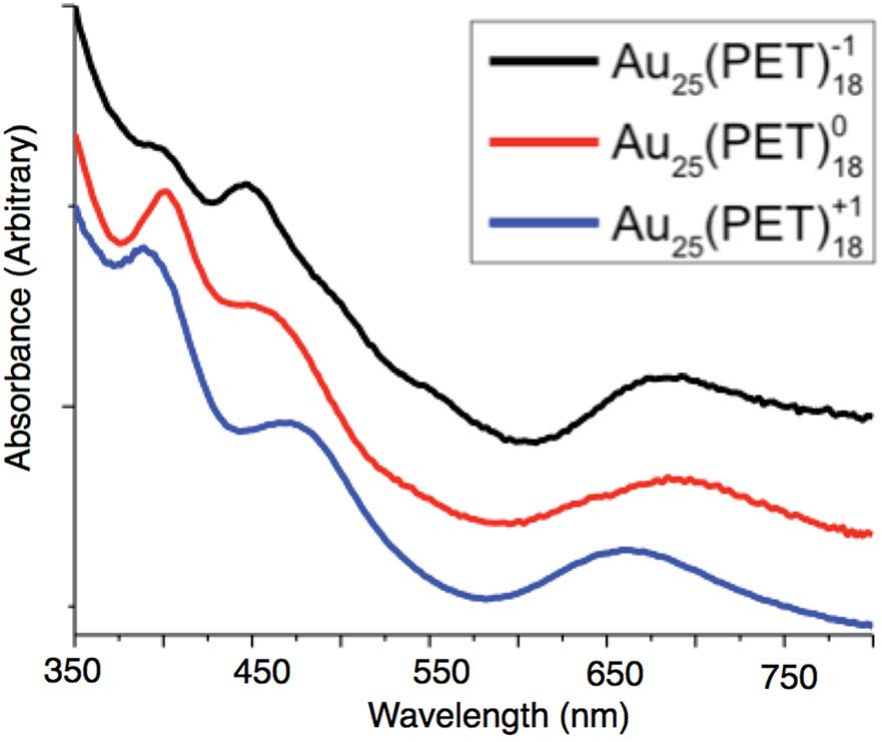
The linear absorption spectra of Au_25_(SR)_18_^–1/0/+1^. The spectra are normalized and offset for clarity.

The experimental and theoretical spectra of Au_25_(PET)_18_ are previously reported.^14^,^19^,^21^,^24^ We improved the experimental spectra for each charge state by forming, isolating and redissolving X-ray quality single crystals of each charge state. We replot our data with previously reported spectroelectrochemical data in Fig. 5 as previously noted, the linear absorption spectrum changes substantially for each oxidation state. We correlate these changes here to changes in the structure of each oxidation state. Relative to Au_25_(PET)_18_^–1^ the 1P to 1D_e_ transition shows a slightly decreased energy gap of about 0.03 eV for Au_25_(PET)_18_^0^, while the energy of this transition increases for Au_25_(PET)_18_^+1^ by approximately 0.1 eV. The decrease in the HOMO–LUMO energy gap from Au_25_(PET)_18_^–1^ to Au_25_(PET)_18_^0^ is due to one of the 1P orbitals increasing in energy, but still being occupied by one electron, depicted in a qualitative energy level diagram in Fig. 3. With the removal of a second electron the splitting of the 1P orbitals becomes much greater than thermal energy, and the highest energy 1P orbital becomes unoccupied. The decrease in the energy gap of the 1P to 1D_t_ going from with increasing oxidation of Au_25_(PET)_18_ can be attributed to the splitting of the superatomic D orbital degeneracy. This first-order Jahn–Teller distortion is reflected in the distortion from ideal symmetry in the crystal structures.^19^,^26^ We suggest that the increase in energy gap for the ligand band to 1D_e_ in Au_25_(PET)_18_^+1^ arises from the electron deficient core pulling electron density from the ligand shell. This is supported by the shorter average bond lengths between sulfur and shell **I** Au atoms in Au_25_(PET)_18_^+1^.

Theoretical optical absorption spectra (Fig. S9†) show a qualitative agreement with the experimental data, particularly showing the systematic blue shift of the first absorption peak as the oxidation state increases from –1 to +1. We calculated the spectrum of +2 state in the experimental configuration of +1. The spectrum is significantly different from +1 spectrum at low excitation energies and confirms that +2 clusters are not impurities in the solution of +1.

### Magnetic properties of Au_25_(SR)_18_^–1/0/+1^

We report the first investigation of magnetism in the Au_25_(SR)_18_^–1/0/+1^ cluster by Superconducting Quantum Interference Device (SQUID). Relative to NMR and EPR approaches SQUID incorporates greater sensitivity, allowing observation of smaller molar magnetic susceptibilities (*χ*_m_). SQUID measures the total susceptibility of a sample whereas previous studies were limited to paramagnetic susceptibility. Subtraction of the diamagnetic contribution from *χ*_m_ allows determination of the paramagnetic susceptibility (*χ*_p_). *χ*_p_ can be used for the comparison of a magnetic moment to that of a free electron. The diamagnetic susceptibilities were approximated from Pascal's diamagnetic corrections.

**Fig. 6:**
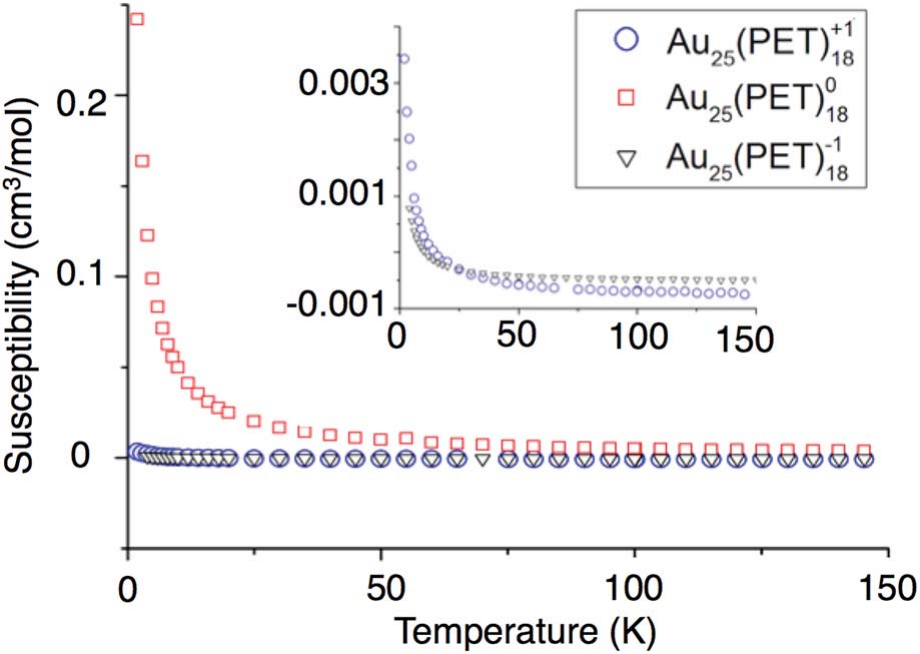
The susceptibilities *versus* temperature of –1 (blue circles), 0 (red squares), and +1 (black triangles) are shown above.

To determine the charge dependent magnetic behavior of Au_25_(SR)_18_, temperature was ramped from 4 K to 300 K under a magnetic field of 0.1 Tesla. In this regime, paramagnetic substances show a response that is inversely proportional to the temperature, and diamagnetic substances show a temperature-independent response. Fig. 6 shows the *χ*_p_*vs.* temperature. We conclude that Au_25_(PET)_18_ in –1 and +1 oxidation states is almost ideally diamagnetic. This observation agrees with the computational prediction for the spin-singlet ground state of Au_25_(PET)_18_^+^ (the spin-triplet state is predicted to be +0.39 eV higher in energy). Deviations from ideal behavior are reflected in a very small paramagnetic-type response, observable only at very low temperatures for –1 and +1. Conversely, Au_25_(PET)_18_ as a neutral compound produces a nearly ideal paramagnetic response that could be observed up to 300 K. The paramagnetic susceptibilities found from SQUID for Au_25_(SR)_18_^–1/0/+1^ correspond to 0.01, 1.07, and 0.03 unpaired electrons, respectively.

These values assume ideal paramagnetic behavior, where magnetic anisotropy or magnetic coupling violate the assumption. To determine magnetic anisotropy, measurements were made at low temperatures (2–32 K) and large magnetic fields (1–5 T). Under these conditions, the unpaired electrons within a paramagnetic substance all align with the external field and response is expected to fit to the Brillouin function of magnetism.^38^ The SQUID data for Au_25_(PET)_18_^0^ fits the Brillouin function for a spin value of 1/2 and a *g*-factor of 2.16, as shown in Fig. S5.† Minimal magnetic anisotropy is thus suggested. Here, the *g*-factor value indicates spin–orbit coupling, similar to a previous conclusion for this system.^23^,^31^

First order Jahn–Teller distortions resulting in the splitting the degeneracy of superatom P orbitals account well for the magnetic behavior of Au_25_(PET)_18_. However, some paramagnetic susceptibility is observed that is not accounted for by this simple approximation. Previous studies have reported that the gold 5d orbital is partially depleted in its bonding to sulfur.^9^,^32^ This may result in a magnetic moment that would correspond to a fraction of an unpaired electron on gold bonded to sulfur, which in the ensemble of an Au_25_(SR)_18_ molecule is observed as a small magnetic moment for the –1 and +1 oxidation states.

Compared to Au_25_(PET)_18_^–1^, Au_25_(PET)_18_^+1^ has a slightly larger magnetic susceptibility. We propose that this arises from greater electron deficiency in Au_25_(PET)_18_^+1^, which pulls electron density inward, creating larger d holes in the semiring Au(i) atoms compared to Au_25_(PET)_18_^–1^. According to the Bader charge analysis, 0.34*e* and 0.28 are depleted from the core and semiring Au atoms, respectively, when comparing Au_25_(PET)_18_^+1^ to Au_25_(PET)_18_^–1^ (Table S6†). The magnetic behavior of Au_25_(PET)_18_^0^ is more complicated. Here we propose that due to the almost degenerate P orbitals, the paramagnetic susceptibility in excess of 1.0 unpaired electrons arises from spin–orbit coupling.^23^,^31^ We estimate that 1–3% of an unpaired electron arises from the Au–S interaction (d-holes), with the remaining (4–6%) arising from superatomic spin–orbit coupling for Au_25_(PET)_18_^0^. Our values for magnetism in the anionic compound are consistent with previously reported results.^32^

### Long range order and packing of Au_25_(PET)_18_PF_6_

**Fig. 7:**
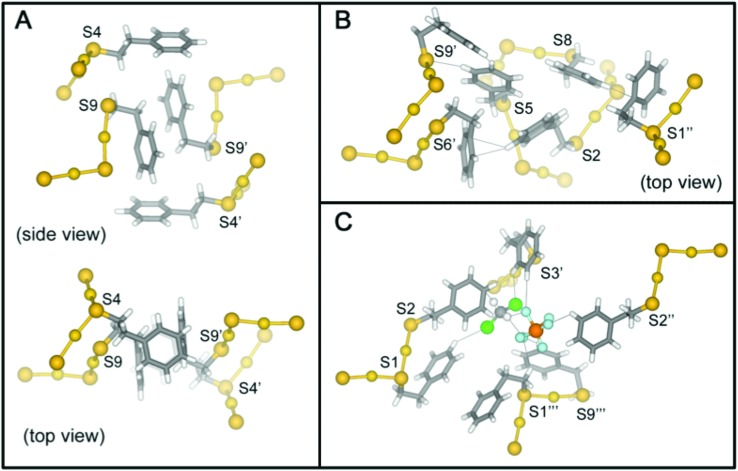
The π-stacking squares formed by PET4 and PET9 of adjacent clusters are shown in panel (A). The extended π-interaction network of PET1, PET2, and PET8 with PET5, PET6 and PET9 of an adjacent cluster are shown in panel (B). The ligands involved in phenyl–halogen and phenyl–solvent interactions important for crystal packing are shown in (C). Ligands of neighboring clusters are denoted by an apostrophe.

The high-quality of the two reported crystal structures prompts the first complete analysis of molecular packing interactions in single-crystals of thiolate protected gold. Indeed, clusters with ligand shells comprised of aromatic ligands such as PET and pMBA account for most crystal structures of ligated gold nanoparticles. In the case of Au_25_(SR)_18_^–1/0/+1^, there are substantial differences in the ligand shell structure in the solid state for each charge state. These differences in the ligand layer do not appear to be propagations of the changes in the inorganic core due to charge state; rather, the differences in the ligand layer of Au_25_(SR)_18_^–1/0/+1^ arise from different inter- and intra-molecular ligand–ligand interactions, ligand–counterion interactions, and ligand–solvent interactions (Fig. 7).

The high quality of the Au_25_(SR)_18_^+1^ structure reported here allows a careful analysis of the role of phenylethane thiolate ligands in the packing of Au_25_(PET)_18_^+1^ into single crystals. To our knowledge, no similar analysis has been previously reported; the interactions described here, however, appear to be ubiquitous among PET protected AuNC structures.^13^,^14^,^26^,^33^–^35^ The importance of this analysis is due to the ligand shell of thiolate protected gold nanoparticles largely determining the interaction of the cluster with its external environment, for instance, in biological contexts.^36^,^37^

Due to the imposed inversion symmetry of the *P*1(bar) space group, there are nine crystallographically independent PET ligands found on the cluster surface (Fig. 1) located in three crystallographically independent semirings (S–Au–S–Au–S units) shown in Fig. S1 and S2.† Table 1 summarizes the dominant intra- and inter-molecular interactions of each of the nine symmetry-unique ligands in the Au_25_(SR)_18_^+1^ crystal structure.

Each ligand adopts either *anti* or *gauche* conformation on the cluster surface, corresponding to an S–CH_2_–CH_2_–Ph torsion angle of ∼180° or ∼60°, respectively (Table 1, Scheme S1, Fig. S1†). Four of the five *gauche* ligands (PET1, PET6, PET7 and PET9) fold over the semiring to which they are bonded and form cation–aromatic interactions with the Au^I^ atom in the semiring. Specifically, Au^I^···π interactions are observed, with average distance of 3.43 Å (Fig. S3†). A fifth *gauche* ligand (PET3) does not form cation–phenyl interaction with the Au^I^ atom in the unit. Instead it coordinates to the PF_6_^–^ counter anion and DCM solvent molecule that sit above the corresponding Au^I^ atom (Au3), preventing the Au^I^···π interactions observed for other *gauche* ligands.

The remaining four crystallographically independent ligands (PET2, PET4, PET5 and PET8) form inter-cluster CH···S, CH···Ph and Ph···Ph interactions with the ligands of adjacent Au_25_ clusters. In addition, these ligands form intermolecular Ph···F, Ph···Cl and CH···F interactions with the PF_6_^–^ anions or DCM solvent molecules within the crystal lattice.

We observe three structural motifs that underlie the intermolecular interactions among adjacent Au_25_(PET)_18_^+1^ clusters. A packing diagram for the crystalline arrangement of clusters is shown in Fig. S3.† The three motifs that mediate this assembly are: (1) phenyl–phenyl′ squares (where the ′ denotes a phenyl ring from a neighboring cluster); (2) an extended π-interaction network involving 6 ligands; (3) halogen mediated interactions of PET–PF_6_–DCM–PET construction. An example of each of these interactions is shown in Fig. 7.

In the phenyl–phenyl′ square assembly, PET9 ligands interact with the respective ligands of the neighboring Au_25_ cluster by forming π···π and CH···π inter-cluster interactions (Fig. 7, panel A). The sides of the square are composed of parallel displaced opposite facing PET9 ligands forming both π···π (3.34 Å) and CH···π (2.69 Å) interactions. The other two sides of the square assembly are defined by PET4 ligands, which form a perpendicular edge-to-face π···π (2.84 Å) interaction with the respective PET9 ligand. A second neighboring Au_25_ cluster additionally interacts with PET4 ligand from the opposite side by forming tilted edge-to-face π···π (2.81 Å) interactions with PET5′ and PET6′ ligands and CH···π (2.85 Å) interaction with PET7′ ligand. Fig. 7 panel A illustrates this assemblage.

The extended π-interaction network is nucleated by three PET ligands (PET2, PET5 and PET8) in the anti-conformation, which are located at the S2–S5–S8 intersection of the three separate semirings (Fig. S2†). These ligands form intermolecular interactions with one another and also interact with the ligands of two neighboring Au_25_ clusters, and also with the PF_6_^–^ anion and DCM solvent molecule (Fig. 7, panel B, DCM solvent not shown). PET2 and PET5 coordinate to one of the adjacent Au_25_ clusters, forming tilted edge-to-face and edge-to-edge π···π (2.80 and 2.38 Å) interactions with the neighboring PET6′ and PET9′ ligands, respectively. In addition to the aromatic interaction, the PET5 ligand quite interestingly also forms PhH···S (2.92 Å) interaction with the sulfur atom of the neighboring PET9′ ligand. The PET8 ligand of the nucleating cluster, on the other hand, connects to a second neighboring Au_25_ cluster by forming perpendicular edge-to-face π···π (2.83 Å) interaction with its PET1′′ ligand. The space between the two neighboring Au_25_ clusters is occupied by the DCM–PF_6_–PF_6_–DCM complex (*vide infra*) and in addition to the prevailing aromatic inter-cluster interactions, PET2 ligand is also available to form π···HC (2.90 Å) and PhH···F (2.60 Å) interactions with the solvent DCM and PF_6_^–^ anion, respectively.

The voids in the distorted simple cubic lattice formed by Au_25_(PET)_18_ nuclei in the single crystal are occupied by a DCM–PF_6_–PF_6_–DCM complex that not only fills the available space, but also coordinates to the neighboring PET ligands (PET1, PET2, PET3 and PET9) forming directional aromatic–halide and aromatic–CH weak inter-cluster interactions (Fig. 7, panel C). As such, one Au_25_ cluster is surrounded by total of six DCM–PF_6_–PF_6_–DCM complexes in the crystal lattice. Due to the directional halide–halide and aromatic–halide intermolecular interactions offered by the DCM–PF_6_–PF_6_–DCM, the complex fills almost perfectly the space between the Au_25_ clusters in the crystal lattice. This seems to have a strong effect on the crystal packing arrangement and gives an extremely good quality crystal structure which is also seen as the lack of disorder in the ligand layer. A more in depth discussion of these interactions is found in the ESI.†

## Conclusions

The determination of the crystal structures of Au_25_(PET)_18_ in three discrete charge states allows for the first time a comparison of electronic and magnetic differences of all three stable charge states of Au_25_(SR)_18_ in the context of their structure. The Jahn–Teller effect is a convenient structural framework to describe the evolution of structure as oxidation state changes. Au_25_(PET)_18_^–1^ has a noble gas-like configuration (1S^2^1P^6^) underlying its diamagnetism and comparatively high thermal stability. Comparatively, Au_25_(PET)_18_^0^ with 1S^2^1P^5^ superatom electron configuration is paramagnetic arising from an unpaired 1P electron. When incomplete, the superatomic 1P become non-degenerate, which is reflected in the structure of the cluster becoming oblate relative to the anion. Oxidation to Au_25_(PET)_18_^+1^ (1S^2^1P^4^) results in larger distortions to the cluster than are observed in either of the other charge states. The electronic distortion results in an unoccupied P orbital in Au_25_(PET)_18_^+1^, rendering it diamagnetic. Here we show for the first time that Jahn–Teller effects apply to thiolate protected gold clusters. The superatom driven distortions are primarily observed in the 13 gold atoms of shell **I**, with subsequent shells reflecting smaller distortions. A Jahn–Teller effect for Au_24_X(SR)_18_ where X = Pd or Pt was recently reported by another group, based on spectroscopic evidence, while this paper was under revision.^39^

## Supplementary Material

Supplementary information

Crystal structure data
