# Supplementary material for: Jahn–Teller effects in Au25(SR)18
Source: Chem Sci. 2015 Nov 24;7(3):1882–90. doi: 10.1039/c5sc02134k (PMC5965251; doi:10.1039/c5sc02134k)
Supplement: Supplementary file 1 [file SC-007-C5SC02134K-s001.pdf]

Electronic Supplementary Material (ESI) for Chemical Science.
This journal is © The Royal Society of Chemistry 2015

Experimental

$[Au_{25}(PET)_{18}]^-$ $[TOA]^+$: $Au_{25}(PET)_{18}^{-1}$ was synthesized by co-dissolving 1.00 g (2.54 mmol) of $HAuCl_4 \cdot 3H_2O$ and 1.56 g (2.85 mmol) of tetraoctylammonium bromide (TOAB) in 70 ml of THF. This solution was allowed to stir for 15 min over which time the solution turns from yellow to orange. Next 1.8 ml (13.4 mmol) of 2-phenylethanethiol (PET) is added to the solution. The reaction mixture was stirred until it turned clear, which takes about 3 hours. Once the solution turned clear a freshly prepared aqueous solution containing 965 mg (25.5 mmol) of $NaBH_4$ and 24 ml of water at 0 C ° was prepared. This aqueous solution was then rapidly added to the THF solution under vigorous stirring and was allowed to stir for 2 days. The reaction mixture was loosely covered to prevent the loss of THF over this course of time. [1]

After 2 days the water is decanted and the THF dried. This gives an oily mixture and any additional water was removed. Methanol is added to the crude product which causes a precipitate to form. It is useful to sonicate at this step to ensure that methanol mixes with all the oil. It is important to note here that $Au_{25}(PET)_{18}^-$ is slightly soluble in sufficiently dry ethanol, and so methanol is preferred. Next the precipitate is removed from the methanol by centrifugation. The precipitate was then washed with methanol 4 more times, or until the odor of thiol no longer remains. Next the precipitate was extracted with dichloromethane (DCM). The DCM was then removed and the product is dissolved in toluene. In order to prevent air oxidation about 100 mg of TOAB was also dissolved in the toluene. It is important to remove most impurities in order to crystallize the product. This can be done by fractional precipitation by adding ethanol to the toluene solution and centrifuging, until the precipitate appears to have some Au_{25} in it as judged by UV/Vis. Any remaining impurities can be removed by the crystallization of $Au_{25}(PET)_{18}^-$ from the toluene:ethanol solution by slow cooling. The yield is 20-30% on gold atom basis.

$Au_{25}(PET)_{18}^0$: $Au_{25}(PET)_{18}^0$ was synthesized by the oxidation of Au_{25}^-. This was done in three different ways. The most common method of oxidation of $Au_{25}(PET)_{18}^{-1}$ to $Au_{25}(PET)_{18}^0$ was done by dissolving crystal pure $Au_{25}(PET)_{18}^-$ in DCM and shaking in the presence of silica gel. This occurs over a short period of time. If the solution is dilute enough one will observe a color change from orange ($Au_{25}(PET)_{18}^{-1}$) to yellow ($Au_{25}(PET)_{18}^0$). (see picture 1)

If the desired product is $Au_{25}(PET)_{18}^0$ and not $Au_{25}(PET)_{18}^-$, then the crude product produced after the removal of THF and water from the $Au_{25}(PET)_{18}^-$ synthesis can be run through a silica gel column using a solvent mixture of 1:1 of DCM:hexanes. This product is than dried. $Au_{25}(PET)_{18}^0$ can then be crystallized by dissolving $Au_{25}(PET)_{18}^0$ in toluene and adding ethanol until it becomes saturated. Crystals are then formed through slow cooling.

This product can also be made through bulk electrolysis in a solution of 0.1 M tetrabutylammonium hexafluorophosphate ($TBAPF_6$) in DCM at a potential of 20 mV vs SCE.

$[Au_{25}(PET)_{18}]^+$ $[PF_6]^-$: $Au_{25}(PET)_{18}^+$ was synthesized by bulk electrolysis from the two times crystallized $Au_{25}(PET)_{18}^-$. $Au_{25}(PET)_{18}^-$ was dissolved in a solution containing 0.1M $TBAPF_6$ in DCM. Bulk electrolysis was preformed at a constant potential in a three-compartment cell at 300 mV vs SCE. Immediately after the bulk electrolysis was complete, the solution was prepared for crystallization, as this compound appears to be unstable in solution for short periods of time. Ethanol was added to the DCM solution used in bulk electrolysis until a precipitate formed. This was than centrifuged and the solution was decanted. This was repeated until the precipitate appears to contain $Au_{25}(PET)_{18}^{+1}$, as judged by UV/Vis. Once this $Au_{25}(PET)_{18}^{+1}$ is sufficiently pure the solution will appear green instead of yellow or orange. At this point the $Au_{25}(PET)_{18}^{+1}$ was put into a freezer at -20 °C with no insulation.

Electrochemical methods

Electrochemistry was preformed using a BAS 100 B potentiostat. All electrochemical techniques were preformed in a DCM solution containing 0.1M $TBAPF_6$ using a standard calomel electrode. Cyclic voltammetry, differential potential voltammetry, and square wave voltammetry were performed using a glassy carbon electrode. Bulk electrolysis was performed using a platinum wire for both the working and counter electrode.

SQUID

85.5 mg, 96.3 mg and 23.6 mg of crystalline $Au_{25}(PET)_{18}$ in the -1, 0 and +1 charge state, respectively, were powdered and loaded into a gel capsule and placed into a straw. These samples were than measured using a DC head. Temperature dependent susceptibility measurements were made using a magnetic field of 1000 Oe.

	$Au_{25}(PET)_{18}^{-1}$	$Au_{25}(PET)_{18}^{0}$	$Au_{25}(PET)_{18}^{+1}$
**S-I**	15.28	22.14	26.36
**Au-II**	2.74	3.73	7.3
**S-III**	3.94	0.38	5.07

Table S1. The average dihedral angle (°) for shells **II**-**IV** with respect to shell **I**.

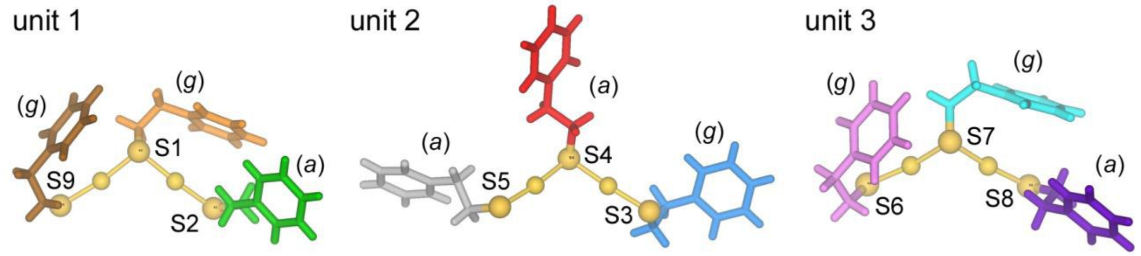

Figure S1. Crystallographically independent semirings, units 1, 2 and 3, showing the *gauche* (g) and *anti* (a) torsion angles of the 9 crystallographically independent PET ligands (see Table 1 for color code).

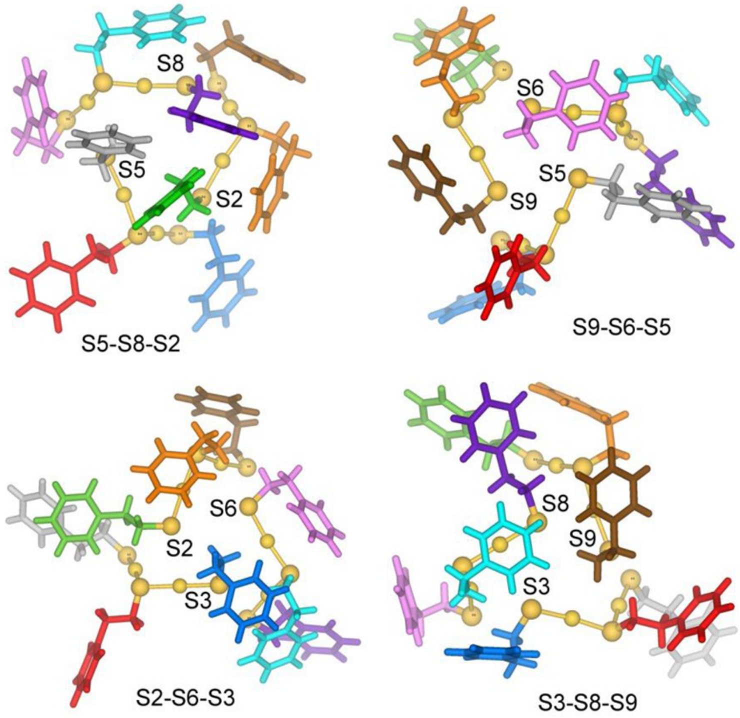

Figure S2. Cluster surface viewed from the four different intersections of the units 1, 2 and 3.

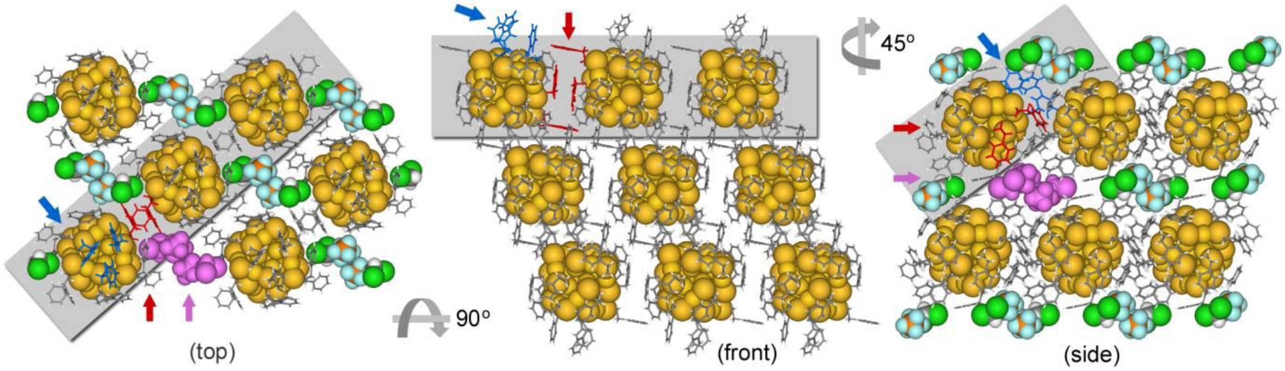

Figure S3. Packing diagrams viewed from top, front and side views highlighting the intercluster interaction themes 1 (**red**), 2 (**blue**) and 3 (**magenta**) between the PET ligands of the neighboring $Au_{25}(PET)_{18}^{+1}$ clusters, counter anion PF_6^- and solvent DCM molecules in the crystal lattice.

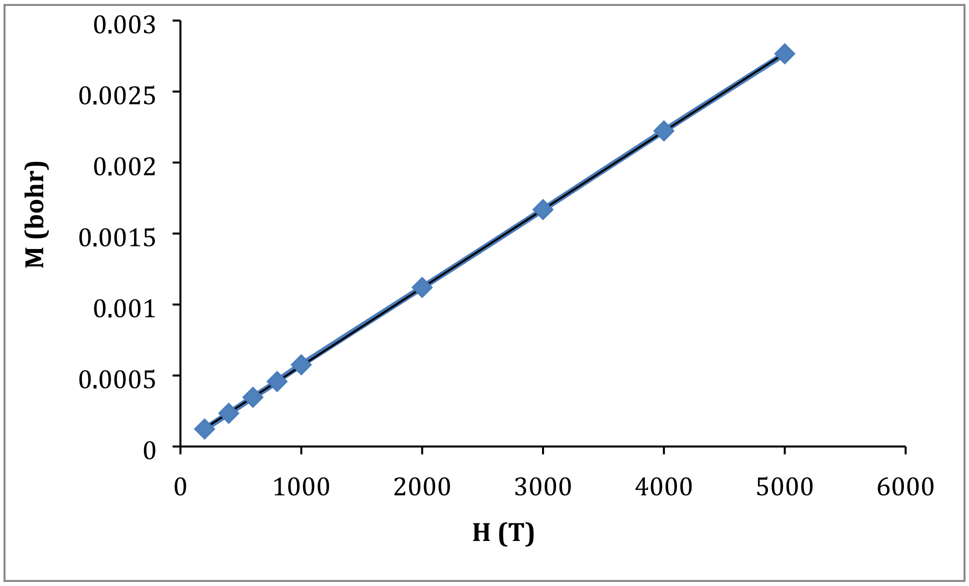

Figure S4. Susceptibility vs magnetic field for $Au_{25}(PET)_{18}^0$. A linear response indicates a typical paramagnetic response, and no ferromagnetic impurities.

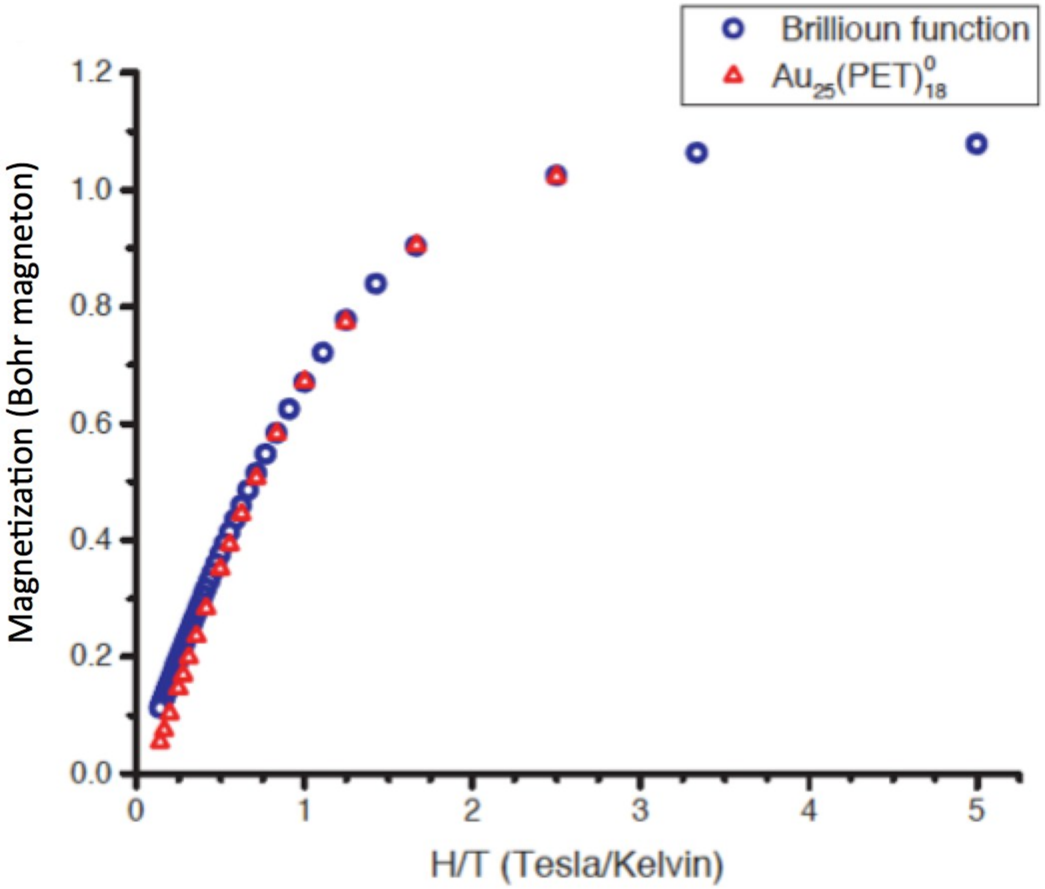

Figure S5. Magnetization of $Au_{25}(PET)_{18}{}^{0}$ compared to the Brillion Function.

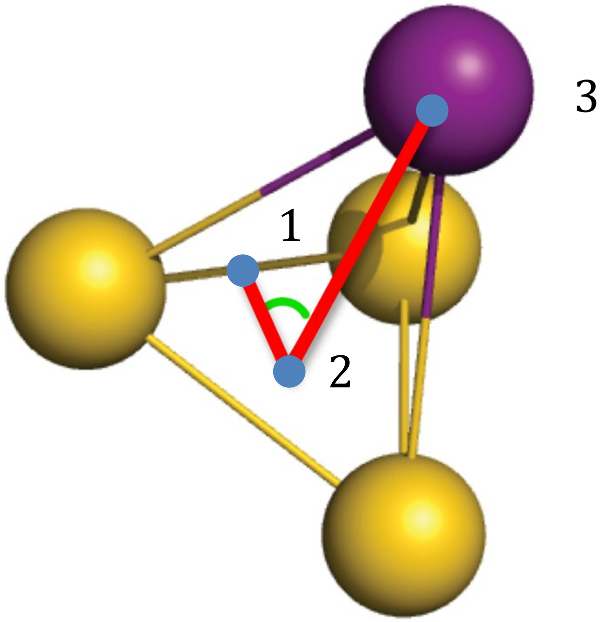

Figure S5: The schematics showing how the bond angles of shell **I-III** were measured. Three points were used: 1) center of the bond in shell **I**, 2) center of the face of shell **I**, and 3) gold atom (purple) of shell **III.** The absolute value obtained by subtracting from 90 ° was averaged for each edge measured and this value was averaged for all 12 Au(I) atoms in shell **III.**

		$Au_{25}(PET)_{18}^{-1}$	$Au_{25}(PET)_{18}^{0}$	$Au_{25}(PET)_{18}^{+1}$
Shell I				
	Average edge bond length	2.92	2.94	2.94
	Variation in edge length	0.3	0.4	0.7
	Standard deviation	0.08	0.11	0.18
bond I-II				
	Average	2.40	2.40	2.38
	Biggest variation in bond length	0	0	0.2
	Standard deviation	0	0	0.06
II-III bond length				
	Average bond length	2.30	2.30	2.30
	Standard deviation	0	0	0
III-IV bond length				
	Average bond length	2.30	2.30	2.30

	Standard deviation	0	0	0

Table S2. Average bond lengths (Å) for $Au_{25}(PET)_{18}$ in all three charge states.

I-II-III angle	$Au_{25}(PET)_{18}^{-1}$	$Au_{25}(PET)_{18}^{0}$	$Au_{25}(PET)_{18}^{+1}$
**sum of both sides ave**	173.7	174.2	180.5
**std**	0.6	1.2	1.34

Table S4. Measured angles (°) for the semi-rings.

	$Au_{25}(PET)_{18}^{-1}$	$Au_{25}(PET)_{18}^{0}$	$Au_{25}(PET)_{18}^{+1}$
**# unpaired e**	0.01	1.07	0.03
**g-factor**	N/A	2.16	N/A

Table S5. Summary of the magnetic properties of $Au_{25}(PET)_{18}^{-1/0/+1}$.

Additional Crystal Packing Analysis of $Au_{25}(PET)_{18}^{+1}$

The DCM-PF_6-PF_6-DCM complex is composed of a coordinating DCM solvent molecule to two fluoride atoms of the PF_6^- anion via CH···F (2.394 Å) intermolecular interactions (Figure S8). The PF_6^- anion then coordinates to the second PF_6^- anion imposed by the P-1 symmetry element via two F···F (2.681 Å) intermolecular halide-halide interactions, which seems to be quite typical interaction of PF_6^- anions in close proximity in the crystal lattice.[4] Due to the P-1 symmetry of the structure, only the interactions of the DCM-PF_6 complex have been considered in the following. The DCM-PF_6 complex is surrounded by 3 neighboring Au_{25} clusters (or 6 when considering full symmetry) and intermolecular interactions are formed to the closest PET ligands (Figure S7). PET1 and PET2 ligands from one Au_{25} cluster coordinate to the DCM solvent molecule via ArH···Cl (2.621 Å) interactions. PET3' ligand of a second neighboring Au_{25} cluster forms ArH···Cl (2.941 Å) and ArH···F (2.590 Å) interactions to both the DCM solvent and the PF_6^- anion in a similar manner to PET1'' and PET9'' ligands of the third neighboring Au_{25} cluster that likewise form CH···F (2.596 Å) and ArH···F (2.602 Å) interactions but only to the PF_6^- anion. The structure contains numerous organized packing interactions, which are most likely due to the coordinating solvent and anion in the crystal lattice that allow the formation of the organized and directional weak intercluster interactions to be formed instead of mere closest packing of the Au_{25} clusters.

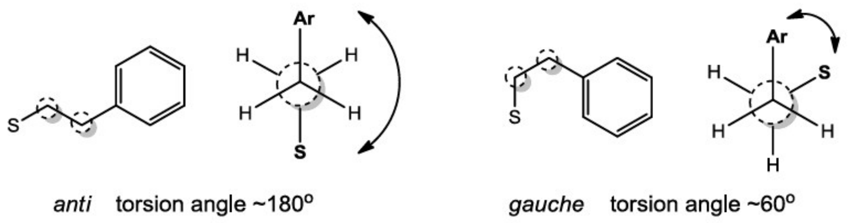

Scheme 1. *Anti* and *gauche* conformations of the PET ligand.

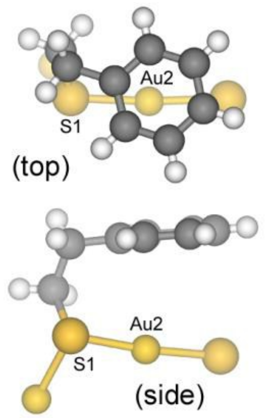

Figure S6. a) Cation-π interactions on the cluster surface shown between the folded PET1 and Au(I) atom (Au2) in the unit from top and side views. Only part of the cluster is shown.

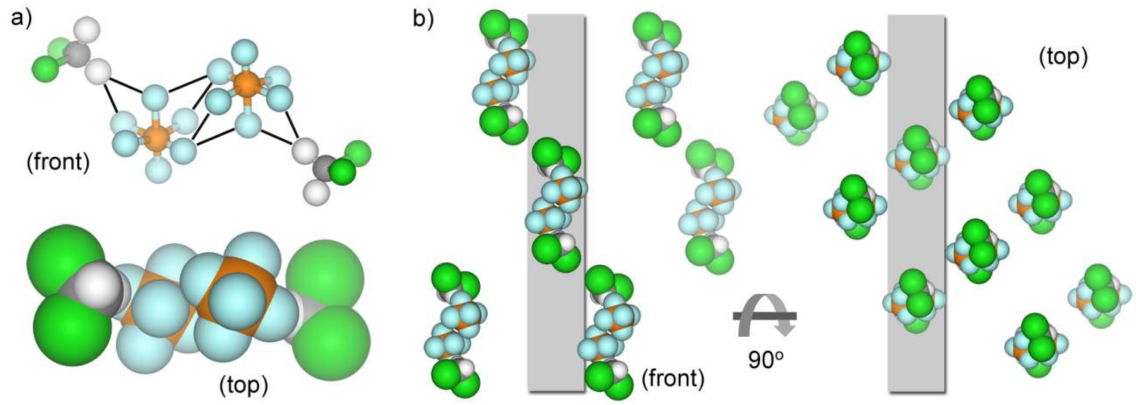

Figure S8. Role of the DCM-PF_6-PF_6-DCM complex in the crystal packing of $Au_{25}(PET)_{18}^{+1}$: a) Intermolecular CH-halide and halide-halide interactions between the DCM solvent molecule and PF_6^- anions and b) space occupied by the DCM-PF_6-PF_6-DCM complex in the crystal lattice.

Extra Uv/Vis

	$Au_{25}(PET)_{18}^{-1}$	$Au_{25}(PET)_{18}^{0}$	$Au_{25}(PET)_{18}^{+1}$
**core**	685 nm	693 nm	660 nm
**homo-lumo+1**	450 nm	463 nm	477 nm
**ligand to D**	402 nm	402 nm	393 nm
**core**	1.81 eV	1.78 eV	1.88 eV
**homo-lumo+1**	2.76 eV	2.68 eV	2.58 eV
**ligand to D**	3.08 eV	3.08 eV	3.15 eV

Table S6 UV/Vis data of $Au_{25}(PET)_{18}^{-1/0/+1}$.

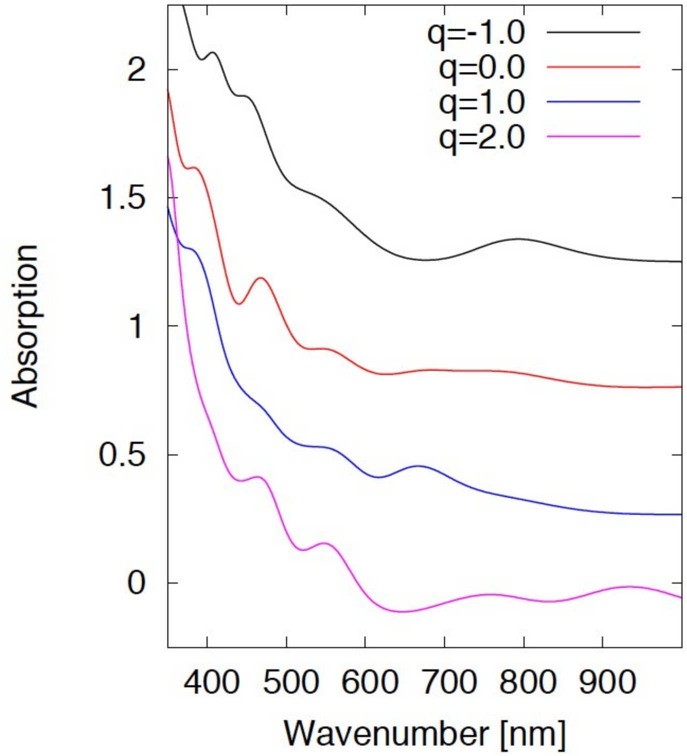

Figure S9: Calculated optical absorption spectra of $Au_{25}(PET)_{18}^{q}$ clusters with charge states q = +2, +1, 0, -1. The curves are offset in the y-axis for clarity.

Atomic layer	N	$Au_{25}(PET)_{18}^{-1}$	$Au_{25}(PET)_{18}^{0}$	$Au_{25}(PET)_{18}^{+1}$
1. Au-atoms of the core	13	0.29	0.48	0.63

2. Au-atoms of the units	12	1.12	1.15	1.40
3. PET-groups	18	-2.42	-1.65	-1.06

Table S6. Bader charges of the atomic layers (in |e|) for $Au_{25}(PET)_{18}^{q}$ cluster with different charge states q = -1, 0, +1.

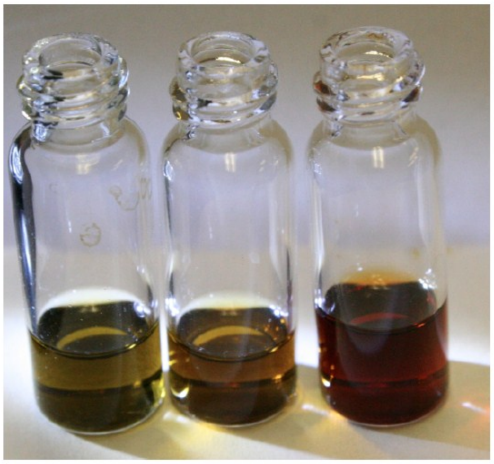

Figure S10. Solutions of $Au_{25}(PET)_{18}$ in the +1(left), 0 (middle) and -1 (right) charge states in dichloromethane.

X-Ray crystallography

X-ray diffraction data from crystals of $Au_{25}(PET)_{18}^{0}$ and $Au_{25}(PET)_{18}^{+1}$ were recorded on a Bruker Nonius SMART CCD diffractometer employing MoK_{α} radiation (graphite monochromator). Selected details related to the crystallographic experiment are listed in Table S2. Unit cell parameters were obtained from a least-squares fit to the angular coordinates of all reflections, and intensities were integrated from a series of frames (0.3º ω rotation) covering more than a hemisphere of reciprocal space. Absorption and other corrections were applied by using SADABS.[2] The structure was solved by using direct methods and refined (on F^2, using all data) by a full-matrix, weighted least-squares process. All non-hydrogen atoms were refined by using anisotropic displacement parameters. Hydrogen atoms were placed in idealized positions and refined by using a riding model. Standard Bruker Nonius control (SMART) and integration (SAINT) software was employed, and Bruker Nonius SHELXTL[3] software was used for structure solution, refinement, and graphics.

Definitions of R_1 and $wR2$.

$R_1 = \Sigma ||F_o| - |F_c|| / \Sigma |F_o|$; $wR2 = \{[illegible][w(F_o^2 - F_c^2)^2] / [illegible][w(F_o^2)^2]\}^1$

	$Au_{25}(PET)_{18}^{0}$	$[Au_{25}(PET)_{18}^{+}(PF_6^{-})]$
**Space group**	$P\bar{1}$	$P\bar{1}$

**Unit cell**	16.1735 x 17.7462 x 17.8295 Å<br>65.00 64.00 81.00 °	16.85 x 17.78 18.24 Å<br>93.76 114.87 112.15 °
**Resolution**	0.73Å	0.75Å
**Wavelength**	MoKα (0.71073Å)	MoKα (0.71073Å)
**# of unique reflections**	23365	22176
**Completeness**	98.1% (93.9%)	99.8% (86.2%)
**<I/σI>**	8.68 (2.49)	15.5 (2.41)
**R_{sym}**	0.1653 (0.4891)	0.1046 (0.3127)
**R**	3.81% (8.91%)	4.53% (7.38%)
**GooF**	1.03	1.044

Table S7. Crystallographic and structure refinement details.

Coordinates for $Au_{25}(PET)_{18}^0$
Au1 Au 0.5000 1.0000 0.5000 0.01559(12) Uani 1 2 d S . P . .
Au2 Au 0.39635(3) 0.85189(2) 0.61796(3) 0.01907(9) Uani 1 1 d . . . . .
Au3 Au 0.42089(3) 0.96788(2) 0.68477(3) 0.01966(9) Uani 1 1 d . . . . .
Au4 Au 0.44735(3) 1.13023(2) 0.55684(3) 0.01964(9) Uani 1 1 d . . . . .
Au5 Au 0.20467(3) 0.86156(3) 0.59211(3) 0.02264(10) Uani 1 1 d . . . . .
Au6 Au 0.77806(3) 0.92738(3) 0.55755(3) 0.02457(10) Uani 1 1 d . . . . .
Au7 Au 0.56794(3) 0.86901(2) 0.61051(3) 0.02087(10) Uani 1 1 d . . . . .
Au8 Au 0.25920(3) 1.08252(3) 0.72870(3) 0.02614(11) Uani 1 1 d . . . . .
Au9 Au 0.61156(3) 1.04428(3) 0.56069(3) 0.02105(10) Uani 1 1 d . . . . .
Au10 Au 0.31085(3) 1.00664(3) 0.58114(3) 0.02098(9) Uani 1 1 d . . . . .
Au11 Au 0.47387(3) 1.09909(3) 0.72886(3) 0.02508(10) Uani 1 1 d . . . . .
Au12 Au 0.42588(3) 0.77284(3) 0.80239(3) 0.02495(10) Uani 1 1 d . . . . .
Au13 Au 0.51021(3) 0.69872(2) 0.62775(3) 0.02188(10) Uani 1 1 d . . . . .
S14 S 0.27622(18) 0.75597(16) 0.6702(2) 0.0221(6) Uani 1 1 d . . . . .
S15 S 0.6304(2) 1.08577(19) 0.6632(2) 0.0291(7) Uani 1 1 d . . . . .
S16 S 0.4427(2) 0.64986(17) 0.7841(2) 0.0258(6) Uani 1 1 d . . . . .
S20 S 0.12149(19) 0.95902(17) 0.5214(2) 0.0274(7) Uani 1 1 d . . . . .
S21 S 0.4105(2) 0.88781(18) 0.8352(2) 0.0311(7) Uani 1 1 d . . . . .
S24 S 0.3201(2) 1.10907(17) 0.8108(2) 0.0278(7) Uani 1 1 d . . . . .
S25 S 0.1830(2) 1.0740(2) 0.6500(3) 0.0386(8) Uani 1 1 d . . . . .
S26 S 0.69559(19) 0.80189(18) 0.6391(2) 0.0265(6) Uani 1 1 d . . . . .
S28 S 0.41337(18) 1.27135(16) 0.5253(2) 0.0230(6) Uani 1 1 d . . . . .
C1_2001 C 0.3001(8) 1.2205(7) 0.7844(10) 0.035(3) Uani 1 1 d . . . . .
H1A_2001 H 0.3144 1.2497 0.7201 0.042 Uiso 1 1 calc R U . . .
H1B_2001 H 0.2356 1.2278 0.8182 0.042 Uiso 1 1 calc R U . . .
C2_2001 C 0.3592(9) 1.2585(7) 0.8087(10) 0.037(3) Uani 1 1 d . . . . .
H2A_2001 H 0.3414 1.2322 0.8738 0.044 Uiso 1 1 calc R U . . .
H2B_2001 H 0.4233 1.2473 0.7787 0.044 Uiso 1 1 calc R U . . .
C3_2001 C 0.3483(8) 1.3506(7) 0.7805(8) 0.031(3) Uani 1 1 d . . . . .
C4_2001 C 0.4235(8) 1.4048(8) 0.7277(7) 0.031(3) Uani 1 1 d . . . . .

H4_2001 H 0.4820 1.3846 0.7057 0.037 Uiso 1 1 calc R U . . .
C5_2001 C 0.4132(9) 1.4901(7) 0.7067(9) 0.037(3) Uani 1 1 d . . . . .
H5_2001 H 0.4642 1.5266 0.6710 0.045 Uiso 1 1 calc R U . . .
C6_2001 C 0.3265(10) 1.5190(8) 0.7394(11) 0.048(4) Uani 1 1 d . . . . .
H6_2001 H 0.3191 1.5758 0.7240 0.058 Uiso 1 1 calc R U . . .
C7_2001 C 0.2503(12) 1.4660(10) 0.7944(16) 0.079(7) Uani 1 1 d . . . . .
H7_2001 H 0.1923 1.4866 0.8176 0.095 Uiso 1 1 calc R U . . .
C8_2001 C 0.2609(10) 1.3811(10) 0.8151(14) 0.074(7) Uani 1 1 d . . . . .
H8_2001 H 0.2097 1.3447 0.8519 0.089 Uiso 1 1 calc R U . . .
C1_2002 C 0.6489(10) 0.9868(9) 0.7488(11) 0.051(4) Uani 1 1 d . . . . .
H1A_2002 H 0.6109 0.9422 0.7608 0.061 Uiso 1 1 calc R U . . .
H1B_2002 H 0.7128 0.9720 0.7253 0.061 Uiso 1 1 calc R U . . .
C2_2002 C 0.6225(14) 0.9983(13) 0.8412(14) 0.075(6) Uiso 1 1 d . . . . .
H2A_2002 H 0.5561 1.0018 0.8699 0.090 Uiso 1 1 calc R U . . .
H2B_2002 H 0.6398 0.9490 0.8822 0.090 Uiso 1 1 calc R U . . .
C3_2002 C 0.664(2) 1.0693(16) 0.8311(15) 0.098(8) Uani 1 1 d . . . . .
C4_2002 C 0.760(2) 1.0781(17) 0.7928(18) 0.150(14) Uani 1 1 d . . . . .
H4_2002 H 0.7947 1.0366 0.7757 0.180 Uiso 1 1 calc R U . . .
C5_2002 C 0.808(2) 1.1494(18) 0.778(2) 0.21(2) Uani 1 1 d . . . . .
H5_2002 H 0.8720 1.1522 0.7530 0.250 Uiso 1 1 calc R U . . .
C6_2002 C 0.759(2) 1.2118(19) 0.802(2) 0.148(13) Uani 1 1 d . . . . .
H6_2002 H 0.7882 1.2592 0.7912 0.178 Uiso 1 1 calc R U . . .
C7_2002 C 0.662(2) 1.1999(16) 0.8447(17) 0.110(9) Uani 1 1 d . . . . .
H7_2002 H 0.6258 1.2384 0.8672 0.132 Uiso 1 1 calc R U . . .
C8_2002 C 0.6176(17) 1.1335(12) 0.8540(13) 0.080(7) Uani 1 1 d . . . . .
H8_2002 H 0.5537 1.1323 0.8768 0.096 Uiso 1 1 calc R U . . .
C1_2003 C 0.4804(7) 1.3188(7) 0.5561(8) 0.027(3) Uani 1 1 d . . . . .
H1A_2003 H 0.4657 1.3771 0.5402 0.033 Uiso 1 1 calc R U . . .
H1B_2003 H 0.4595 1.2929 0.6217 0.033 Uiso 1 1 calc R U . . .
C2_2003 C 0.5838(7) 1.3139(7) 0.5140(8) 0.024(2) Uani 1 1 d . . . . .
H2A_2003 H 0.6067 1.3370 0.4482 0.029 Uiso 1 1 calc R U . . .
H2B_2003 H 0.6011 1.2563 0.5342 0.029 Uiso 1 1 calc R U . . .
C3_2003 C 0.6258(7) 1.3627(8) 0.5424(9) 0.034(3) Uani 1 1 d . . . . .
C4_2003 C 0.6344(8) 1.4482(8) 0.4971(10) 0.043(4) Uani 1 1 d . . . . .
H4_2003 H 0.6139 1.4740 0.4512 0.051 Uiso 1 1 calc R U . . .
C5_2003 C 0.6727(9) 1.4964(10) 0.5184(13) 0.055(5) Uani 1 1 d . . . . .
H5_2003 H 0.6786 1.5538 0.4866 0.066 Uiso 1 1 calc R U . . .
C6_2003 C 0.7018(11) 1.4585(13) 0.5869(14) 0.066(6) Uani 1 1 d . . . . .
H6_2003 H 0.7270 1.4906 0.6022 0.079 Uiso 1 1 calc R U . . .
C7_2003 C 0.6941(11) 1.3737(12) 0.6332(12) 0.058(5) Uani 1 1 d . . . . .
H7_2003 H 0.7142 1.3481 0.6794 0.069 Uiso 1 1 calc R U . . .
C8_2003 C 0.6555(9) 1.3258(10) 0.6098(10) 0.041(4) Uani 1 1 d . . . . .
H8_2003 H 0.6503 1.2682 0.6409 0.050 Uiso 1 1 calc R U . . .
C1_2004 C 0.2974(10) 0.8818(8) 0.9271(9) 0.043(4) Uani 1 1 d . . . . .
H1A_2004 H 0.2990 0.8414 0.9838 0.052 Uiso 1 1 calc R U . . .
H1B_2004 H 0.2860 0.9354 0.9315 0.052 Uiso 1 1 calc R U . . .

C2_2004 C 0.2225(11) 0.8600(10) 0.9187(13) 0.057(4) Uani 1 1 d . . . . .
H2A_2004 H 0.2322 0.8064 0.9144 0.068 Uiso 1 1 calc R U . . .
H2B_2004 H 0.2177 0.9010 0.8638 0.068 Uiso 1 1 calc R U . . .
C3_2004 C 0.1307(11) 0.8558(10) 1.0046(12) 0.064(5) Uani 1 1 d . . . . .
C4_2004 C 0.1207(15) 0.7939(11) 1.0897(15) 0.118(10) Uani 1 1 d . . . . .
H4_2004 H 0.1685 0.7580 1.0942 0.142 Uiso 1 1 calc R U . . .
C5_2004 C 0.0380(17) 0.7850(15) 1.170(2) 0.27(3) Uani 1 1 d . . . . .
H5_2004 H 0.0324 0.7438 1.2259 0.323 Uiso 1 1 calc R U . . .
C6_2004 C -0.0291(14) 0.8350(13) 1.1638(18) 0.096(8) Uani 1 1 d . . . . .
H6_2004 H -0.0840 0.8308 1.2142 0.115 Uiso 1 1 calc R U . . .
C7_2004 C -0.0153(15) 0.8886(17) 1.0864(19) 0.132(12) Uani 1 1 d . . . . .
H7_2004 H -0.0628 0.9248 1.0820 0.158 Uiso 1 1 calc R U . . .
C8_2004 C 0.0636(15) 0.9013(15) 1.0047(18) 0.151(14) Uani 1 1 d . . . . .
H8_2004 H 0.0656 0.9438 0.9506 0.181 Uiso 1 1 calc R U . . .
C1_2005 C 0.2073(7) 0.7326(7) 0.7909(8) 0.025(3) Uani 1 1 d . . . . .
H1A_2005 H 0.2410 0.6972 0.8260 0.030 Uiso 1 1 calc R U . . .
H1B_2005 H 0.1948 0.7838 0.8008 0.030 Uiso 1 1 calc R U . . .
C2_2005 C 0.1169(9) 0.6887(9) 0.8222(10) 0.047(4) Uani 1 1 d . . . . .
H2A_2005 H 0.0796 0.7268 0.7930 0.056 Uiso 1 1 calc R U . . .
H2B_2005 H 0.1293 0.6417 0.8050 0.056 Uiso 1 1 calc R U . . .
C3_2005 C 0.0655(8) 0.6590(10) 0.9247(10) 0.050(4) Uani 1 1 d . . . . .
C4_2005 C 0.0182(9) 0.7153(14) 0.9644(15) 0.21(2) Uani 1 1 d . . . . .
H4_2005 H 0.0144 0.7700 0.9268 0.248 Uiso 1 1 calc R U . . .
C5_2005 C -0.0234(11) 0.6918(13) 1.0584(15) 0.089(7) Uani 1 1 d . . . . .
H5_2005 H -0.0545 0.7294 1.0845 0.107 Uiso 1 1 calc R U . . .
C6_2005 C -0.0154(10) 0.6071(12) 1.1122(12) 0.069(5) Uani 1 1 d . . . . .
H6_2005 H -0.0423 0.5881 1.1753 0.083 Uiso 1 1 calc R U . . .
C7_2005 C 0.0313(10) 0.5535(10) 1.0723(11) 0.055(4) Uani 1 1 d . . . . .
H7_2005 H 0.0368 0.4982 1.1074 0.065 Uiso 1 1 calc R U . . .
C8_2005 C 0.0683(10) 0.5818(9) 0.9830(11) 0.057(5) Uani 1 1 d . . . . .
H8_2005 H 0.0998 0.5439 0.9577 0.068 Uiso 1 1 calc R U . . .
C1_2006 C 0.5368(9) 0.6019(7) 0.8166(10) 0.034(3) Uani 1 1 d . . . . .
H1A_2006 H 0.5862 0.6431 0.7877 0.041 Uiso 1 1 calc R U . . .
H1B_2006 H 0.5152 0.5826 0.8819 0.041 Uiso 1 1 calc R U . . .
C2_2006 C 0.5733(8) 0.5295(7) 0.7888(9) 0.030(3) Uani 1 1 d . . . . .
H2A_2006 H 0.5975 0.5493 0.7231 0.036 Uiso 1 1 calc R U . . .
H2B_2006 H 0.5234 0.4895 0.8154 0.036 Uiso 1 1 calc R U . . .
C3_2006 C 0.6492(9) 0.4869(7) 0.8197(10) 0.035(3) Uani 1 1 d . . . . .
C4_2006 C 0.6300(11) 0.4247(8) 0.9035(11) 0.056(5) Uani 1 1 d . . . . .
H4_2006 H 0.5687 0.4090 0.9432 0.067 Uiso 1 1 calc R U . . .
C5_2006 C 0.6975(16) 0.3841(12) 0.9320(17) 0.096(8) Uani 1 1 d . . . . .
H5_2006 H 0.6829 0.3415 0.9894 0.115 Uiso 1 1 calc R U . . .
C6_2006 C 0.7849(15) 0.4085(12) 0.874(2) 0.091(8) Uani 1 1 d . . . . .
H6_2006 H 0.8314 0.3825 0.8919 0.109 Uiso 1 1 calc R U . . .
C7_2006 C 0.8091(11) 0.4708(13) 0.7872(16) 0.073(6) Uani 1 1 d . . . . .
H7_2006 H 0.8705 0.4860 0.7476 0.088 Uiso 1 1 calc R U . . .

C8_2006 C 0.7385(9) 0.5100(10) 0.7614(11) 0.045(4) Uani 1 1 d . . . . .
H8_2006 H 0.7526 0.5523 0.7039 0.054 Uiso 1 1 calc R U . . .
C1_2007 C 0.0889(10) 0.9760(15) 0.7464(15) 0.084(8) Uani 1 1 d . . . . .
H1A_2007 H 0.1049 0.9258 0.7347 0.101 Uiso 1 1 calc R U . . .
H1B_2007 H 0.0790 0.9641 0.8082 0.101 Uiso 1 1 calc R U . . .
C2_2007 C 0.019(2) 1.0139(13) 0.7270(18) 0.128(12) Uani 1 1 d . . . . .
H2A_2007 H 0.0275 1.0245 0.6660 0.154 Uiso 1 1 calc R U . . .
H2B_2007 H 0.0025 1.0642 0.7384 0.154 Uiso 1 1 calc R U . . .
C3_2007 C -0.0547(17) 0.9297(15) 0.8102(14) 0.163(16) Uani 1 1 d . . . . .
C4_2007 C -0.1119(14) 0.9247(13) 0.8961(12) 0.160(16) Uani 1 1 d . . . . .
H4_2007 H -0.0995 0.9598 0.9169 0.192 Uiso 1 1 calc R U . . .
C5_2007 C -0.1829(12) 0.8729(11) 0.9503(10) 0.069(6) Uani 1 1 d . . . . .
H5_2007 H -0.2193 0.8710 1.0085 0.083 Uiso 1 1 calc R U . . .
C6_2007 C -0.2029(10) 0.8227(11) 0.9214(12) 0.059(5) Uani 1 1 d . . . . .
H6_2007 H -0.2550 0.7874 0.9586 0.070 Uiso 1 1 calc R U . . .
C7_2007 C -0.1480(10) 0.8233(11) 0.8387(13) 0.061(5) Uani 1 1 d . . . . .
H7_2007 H -0.1609 0.7868 0.8198 0.073 Uiso 1 1 calc R U . . .
C8_2007 C -0.0738(12) 0.8767(10) 0.7826(12) 0.062(5) Uani 1 1 d . . . . .
H8_2007 H -0.0362 0.8770 0.7254 0.075 Uiso 1 1 calc R U . . .
C1_2008 C 0.1197(8) 0.9267(7) 0.4370(8) 0.025(2) Uiso 1 1 d . . . . .
H1A_2008 H 0.1108 0.9750 0.3888 0.031 Uiso 1 1 calc R U . . .
H1B_2008 H 0.1786 0.9041 0.4102 0.031 Uiso 1 1 calc R U . . .
C2_2008 C 0.0442(8) 0.8622(7) 0.4783(10) 0.033(3) Uani 1 1 d . . . . .
H2A_2008 H -0.0140 0.8849 0.5064 0.039 Uiso 1 1 calc R U . . .
H2B_2008 H 0.0419 0.8527 0.4295 0.039 Uiso 1 1 calc R U . . .
C3_2008 C 0.0529(8) 0.7795(8) 0.5480(10) 0.032(3) Uani 1 1 d . . . . .
C4_2008 C 0.1264(9) 0.7297(7) 0.5232(10) 0.036(3) Uani 1 1 d . . . . .
H4_2008 H 0.1724 0.7487 0.4643 0.043 Uiso 1 1 calc R U . . .
C5_2008 C 0.1309(8) 0.6512(8) 0.5868(10) 0.041(4) Uani 1 1 d . . . . .
H5_2008 H 0.1804 0.6182 0.5702 0.049 Uiso 1 1 calc R U . . .
C6_2008 C 0.0635(9) 0.6221(8) 0.6735(11) 0.043(4) Uani 1 1 d . . . . .
H6_2008 H 0.0657 0.5692 0.7156 0.051 Uiso 1 1 calc R U . . .
C7_2008 C -0.0077(10) 0.6731(10) 0.6969(11) 0.049(4) Uani 1 1 d . . . . .
H7_2008 H -0.0533 0.6543 0.7561 0.059 Uiso 1 1 calc R U . . .
C8_2008 C -0.0135(8) 0.7507(8) 0.6356(9) 0.034(3) Uani 1 1 d . . . . .
H8_2008 H -0.0624 0.7839 0.6534 0.041 Uiso 1 1 calc R U . . .
C1_2009 C 0.7412(7) 0.7476(7) 0.5614(9) 0.026(3) Uani 1 1 d . . . . .
H1A_2009 H 0.7077 0.6949 0.5901 0.032 Uiso 1 1 calc R U . . .
H1B_2009 H 0.7326 0.7809 0.5062 0.032 Uiso 1 1 calc R U . . .
C2_2009 C 0.8416(8) 0.7325(8) 0.5380(10) 0.032(3) Uani 1 1 d . . . . .
H2A_2009 H 0.8501 0.7027 0.5938 0.039 Uiso 1 1 calc R U . . .
H2B_2009 H 0.8749 0.7856 0.5063 0.039 Uiso 1 1 calc R U . . .
C3_2009 C 0.8818(8) 0.6832(7) 0.4793(10) 0.034(3) Uani 1 1 d . . . . .
C4_2009 C 0.9631(11) 0.6465(10) 0.4728(13) 0.079(7) Uani 1 1 d . . . . .
H4_2009 H 0.9927 0.6524 0.5046 0.094 Uiso 1 1 calc R U . . .
C5_2009 C 1.0031(13) 0.6008(14) 0.4208(17) 0.129(12) Uani 1 1 d . . . . .

H5_2009 H 1.0601 0.5779 0.4158 0.155 Uiso 1 1 calc R U . . .
C6_2009 C 0.9587(12) 0.5888(14) 0.3760(17) 0.104(9) Uani 1 1 d . . . . .
H6_2009 H 0.9831 0.5538 0.3453 0.125 Uiso 1 1 calc R U . . .
C7_2009 C 0.8802(10) 0.6274(11) 0.3763(13) 0.061(5) Uani 1 1 d . . . . .
H7_2009 H 0.8527 0.6225 0.3424 0.073 Uiso 1 1 calc R U . . .
C8_2009 C 0.8405(10) 0.6749(9) 0.4283(11) 0.047(4) Uani 1 1 d . . . . .
H8_2009 H 0.7859 0.7015 0.4292 0.056 Uiso 1 1 calc R U . . .
C9_2010 C 0.5508(10) 0.7504(12) 0.9708(11) 0.066(5) Uani 1 1 d . . . . .
H9_2010 H 0.5060 0.7902 0.9743 0.079 Uiso 1 1 calc R U . . .
C10_2010 C 0.6083(11) 0.7454(11) 0.8894(12) 0.059(5) Uani 1 1 d . . . . .
H10_2010 H 0.6011 0.7816 0.8369 0.071 Uiso 1 1 calc R U . . .
C11_2010 C 0.6768(10) 0.6877(10) 0.8834(10) 0.044(4) Uani 1 1 d . . . . .
H11_2010 H 0.7167 0.6867 0.8273 0.053 Uiso 1 1 calc R U . . .
C12_2010 C 0.6849(10) 0.6319(9) 0.9615(11) 0.045(4) Uani 1 1 d . . . . .
H12_2010 H 0.7297 0.5921 0.9584 0.054 Uiso 1 1 calc R U . . .
C13_2010 C 0.7642(16) 0.5705(13) 0.9571(15) 0.099(8) Uani 1 1 d . . . . .
H13A_2010 H 0.7986 0.5754 0.8955 0.148 Uiso 1 1 calc R U . . .
H13B_2010 H 0.7392 0.5147 0.9954 0.148 Uiso 1 1 calc R U . . .
H13C_2010 H 0.8040 0.5832 0.9776 0.148 Uiso 1 1 calc R U . . .
C14_2010 C 0.6277(10) 0.6348(9) 1.0434(10) 0.047(4) Uani 1 1 d . . . . .
H14_2010 H 0.6329 0.5968 1.0962 0.057 Uiso 1 1 calc R U . . .
C15_2010 C 0.5624(11) 0.6939(11) 1.0476(12) 0.066(5) Uani 1 1 d . . . . .
H15_2010 H 0.5246 0.6961 1.1037 0.079 Uiso 1 1 calc R U . . .

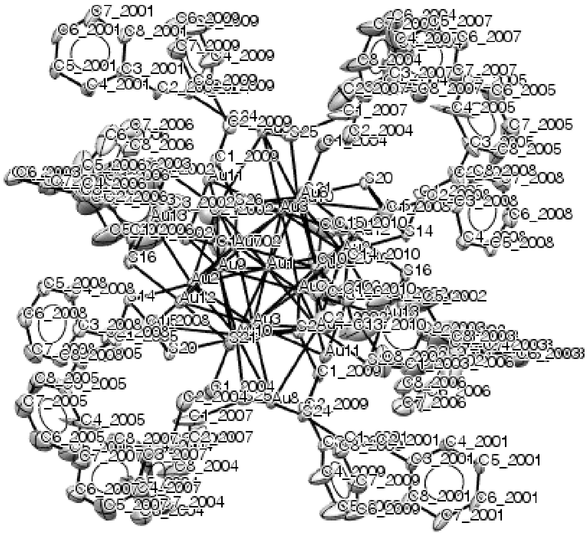

Coordinates for $Au_{25}(PET)_{18}^{+1}$

Au1 Au 1.0000 0.5000 0.5000 0.01142(13) Uani 1 2 d S . P . .
Au2 Au 1.13667(4) 0.52373(3) 0.31115(3) 0.01942(11) Uani 1 1 d . . . . .
Au3 Au 1.22037(4) 0.72330(3) 0.43372(3) 0.01889(11) Uani 1 1 d . . . . .
Au4 Au 1.02055(4) 0.57672(3) 0.37436(3) 0.01683(11) Uani 1 1 d . . . . .
Au5 Au 0.82033(4) 0.43474(4) 0.50142(4) 0.02214(12) Uani 1 1 d . . . . .
Au6 Au 0.82055(4) 0.59765(3) 0.25986(3) 0.02111(12) Uani 1 1 d . . . . .
Au7 Au 0.82533(4) 0.44666(3) 0.34578(3) 0.01691(11) Uani 1 1 d . . . . .
Au8 Au 0.94819(4) 0.22576(3) 0.38356(3) 0.01826(11) Uani 1 1 d . . . . .
Au9 Au 0.76371(4) 0.26812(3) 0.23645(3) 0.01932(11) Uani 1 1 d . . . . .
Au10 Au 0.97251(4) 0.39823(3) 0.35876(3) 0.01879(11) Uani 1 1 d . . . . .
Au11 Au 0.69007(4) 0.51761(3) 0.36896(3) 0.01983(11) Uani 1 1 d . . . . .
Au12 Au 0.87272(4) 0.32576(3) 0.44208(4) 0.02217(12) Uani 1 1 d . . . . .
Au13 Au 0.91191(4) 0.60288(3) 0.44883(4) 0.02604(13) Uani 1 1 d . . . . .
S1 S 0.8022(3) 0.1581(2) 0.2604(2) 0.0222(7) Uani 1 1 d . . . . .
S3 S 0.6988(3) 0.4763(2) 0.4877(2) 0.0212(7) Uani 1 1 d . . . . .
S2 S 1.0952(3) 0.2765(2) 0.5049(2) 0.0207(7) Uani 1 1 d . . . . .
S4 S 0.6721(3) 0.5684(2) 0.2527(2) 0.0234(7) Uani 1 1 d . . . . .
S5 S 0.9623(3) 0.6263(2) 0.2530(2) 0.0210(7) Uani 1 1 d . . . . .
S6 S 0.7276(2) 0.3773(2) 0.1994(2) 0.0210(7) Uani 1 1 d . . . . .
S7 S 1.2537(3) 0.6594(2) 0.3447(2) 0.0208(7) Uani 1 1 d . . . . .
S8 S 1.0268(2) 0.3830(2) 0.2627(2) 0.0201(7) Uani 1 1 d . . . . .

S9 S 1.1926(3) 0.7989(2) 0.5189(2) 0.0215(7) Uani 1 1 d . . . . .
C1 C 1.0593(11) 0.2115(9) 0.5692(10) 0.028(3) Uani 1 1 d . . . . .
H1A H 1.1074 0.2398 0.6291 0.033 Uiso 1 1 calc R U . . .
H1B H 0.9948 0.2052 0.5606 0.033 Uiso 1 1 calc R U . . .
C2 C 1.1079(11) 0.3330(9) 0.3014(10) 0.024(3) Uani 1 1 d . . . . .
H2A H 1.1620 0.3669 0.3583 0.029 Uiso 1 1 calc R U . . .
H2B H 1.0711 0.2763 0.3052 0.029 Uiso 1 1 calc R U . . .
C3 C 0.9231(11) 0.5364(10) 0.1685(10) 0.028(3) Uani 1 1 d . . . . .
H3A H 0.9813 0.5300 0.1746 0.034 Uiso 1 1 calc R U . . .
H3B H 0.8793 0.4843 0.1745 0.034 Uiso 1 1 calc R U . . .
C4 C 0.6726(12) 0.6702(11) 0.2795(11) 0.032(4) Uani 1 1 d . . . . .
H4A H 0.7298 0.7049 0.3348 0.039 Uiso 1 1 calc R U . . .
H4B H 0.6776 0.7010 0.2371 0.039 Uiso 1 1 calc R U . . .
C5 C 0.5778(12) 0.6549(11) 0.2819(13) 0.037(4) Uani 1 1 d . . . . .
H5A H 0.5214 0.6144 0.2287 0.045 Uiso 1 1 calc R U . . .
H5B H 0.5768 0.6288 0.3280 0.045 Uiso 1 1 calc R U . . .
C6 C 0.5658(11) 0.7341(10) 0.2944(11) 0.029(3) Uani 1 1 d . . . . .
H6 H 0.6374 0.7693 0.3162 0.035 Uiso 1 1 calc R U . . .
C7 C 0.5735(14) 0.7698(11) 0.3677(11) 0.037(4) Uani 1 1 d . . . . .
H7 H 0.5899 0.7454 0.4134 0.044 Uiso 1 1 calc R U . . .
C8 C 0.5587(16) 0.8378(12) 0.3770(13) 0.047(5) Uani 1 1 d . . . . .
H8 H 0.5650 0.8607 0.4288 0.057 Uiso 1 1 calc R U . . .
C9 C 0.5343(13) 0.8748(11) 0.3123(14) 0.043(5) Uani 1 1 d . . . . .
H9 H 0.5229 0.9226 0.3191 0.052 Uiso 1 1 calc R U . . .
C10 C 0.5265(15) 0.8420(12) 0.2375(12) 0.043(4) Uani 1 1 d . . . . .
H10 H 0.5086 0.8665 0.1921 0.051 Uiso 1 1 calc R U . . .
C11 C 0.5446(15) 0.7740(12) 0.2292(11) 0.041(4) Uani 1 1 d . . . . .
H11 H 0.5429 0.7533 0.1789 0.049 Uiso 1 1 calc R U . . .
C12 C 0.5854(10) 0.3779(10) 0.4545(9) 0.023(3) Uani 1 1 d . . . . .
H12A H 0.5760 0.3369 0.4085 0.028 Uiso 1 1 calc R U . . .
H12B H 0.5916 0.3525 0.5021 0.028 Uiso 1 1 calc R U . . .
C13 C 0.4975(11) 0.3958(10) 0.4249(10) 0.026(3) Uani 1 1 d . . . . .
H13A H 0.4879 0.4158 0.3739 0.031 Uiso 1 1 calc R U . . .
H13B H 0.5106 0.4415 0.4688 0.031 Uiso 1 1 calc R U . . .
C14 C 0.4053(11) 0.3199(10) 0.4058(9) 0.022(3) Uani 1 1 d . . . . .
H14 H 0.3660 0.3417 0.3628 0.027 Uiso 1 1 calc R U . . .
C15 C 0.3550(11) 0.3210(11) 0.4492(10) 0.029(3) Uani 1 1 d . . . . .
H15 H 0.3802 0.3691 0.4931 0.035 Uiso 1 1 calc R U . . .
C16 C 0.2673(13) 0.2514(13) 0.4283(12) 0.040(5) Uani 1 1 d . . . . .
H16 H 0.2300 0.2545 0.4546 0.048 Uiso 1 1 calc R U . . .
C17 C 0.2342(14) 0.1790(13) 0.3708(15) 0.048(5) Uani 1 1 d . . . . .
H17 H 0.1768 0.1308 0.3601 0.057 Uiso 1 1 calc R U . . .
C18 C 0.3694(12) 0.2473(11) 0.3442(11) 0.034(4) Uani 1 1 d . . . . .
H18 H 0.4025 0.2464 0.3131 0.041 Uiso 1 1 calc R U . . .
C19 C 0.5997(11) 0.3475(10) 0.1638(9) 0.026(3) Uani 1 1 d . . . . .
H19A H 0.5902 0.3992 0.1631 0.032 Uiso 1 1 calc R U . . .

H19B H 0.5615 0.3117 0.1054 0.032 Uiso 1 1 calc R U . . .
C20 C 0.5582(11) 0.3006(10) 0.2157(10) 0.026(3) Uani 1 1 d . . . . .
H20A H 0.5963 0.3353 0.2746 0.031 Uiso 1 1 calc R U . . .
H20B H 0.5646 0.2475 0.2150 0.031 Uiso 1 1 calc R U . . .
C21 C 0.4501(11) 0.2803(10) 0.1821(9) 0.028(3) Uani 1 1 d . . . . .
H21 H 0.4560 0.2758 0.2383 0.034 Uiso 1 1 calc R U . . .
C22 C 0.4245(13) 0.3455(11) 0.1863(11) 0.032(4) Uani 1 1 d . . . . .
H22 H 0.4736 0.4022 0.2113 0.038 Uiso 1 1 calc R U . . .
C23 C 0.3260(13) 0.3273(12) 0.1538(12) 0.040(4) Uani 1 1 d . . . . .
H23 H 0.3084 0.3720 0.1555 0.049 Uiso 1 1 calc R U . . .
C24 C 1.1499(11) 0.3252(10) 0.2433(10) 0.026(3) Uani 1 1 d . . . . .
H24A H 1.1889 0.2938 0.2634 0.032 Uiso 1 1 calc R U . . .
H24B H 1.0952 0.2920 0.1864 0.032 Uiso 1 1 calc R U . . .
C25 C 1.2125(11) 0.4075(9) 0.2380(9) 0.022(3) Uani 1 1 d . . . . .
H25 H 1.2429 0.3797 0.2163 0.027 Uiso 1 1 calc R U . . .
C26 C 1.3063(11) 0.4621(10) 0.3069(10) 0.027(3) Uani 1 1 d . . . . .
H26 H 1.3297 0.4448 0.3567 0.033 Uiso 1 1 calc R U . . .
C27 C 1.3634(13) 0.5389(12) 0.3030(11) 0.036(4) Uani 1 1 d . . . . .
H27 H 1.4251 0.5745 0.3502 0.043 Uiso 1 1 calc R U . . .
C28 C 1.3317(12) 0.5651(11) 0.2306(11) 0.031(4) Uani 1 1 d . . . . .
H28 H 1.3723 0.6181 0.2281 0.038 Uiso 1 1 calc R U . . .
C29 C 1.2400(14) 0.5139(11) 0.1611(11) 0.035(4) Uani 1 1 d . . . . .
H29 H 1.2173 0.5320 0.1117 0.043 Uiso 1 1 calc R U . . .
C30 C 1.1816(11) 0.4341(10) 0.1660(10) 0.026(3) Uani 1 1 d . . . . .
H30 H 1.1199 0.3984 0.1187 0.031 Uiso 1 1 calc R U . . .
C31 C 1.3133(10) 0.8734(8) 0.6033(8) 0.019(3) Uani 1 1 d . . . . .
H31A H 1.3433 0.9187 0.5810 0.023 Uiso 1 1 calc R U . . .
H31B H 1.3053 0.8995 0.6477 0.023 Uiso 1 1 calc R U . . .
C32 C 1.3812(10) 0.8361(9) 0.6416(9) 0.023(3) Uani 1 1 d . . . . .
H32A H 1.3903 0.8104 0.5977 0.028 Uiso 1 1 calc R U . . .
H32B H 1.3517 0.7907 0.6642 0.028 Uiso 1 1 calc R U . . .
C33 C 1.4816(10) 0.9018(9) 0.7127(9) 0.023(3) Uani 1 1 d . . . . .
H33 H 1.4939 0.8567 0.7384 0.027 Uiso 1 1 calc R U . . .
C35 C 1.6540(11) 0.9882(11) 0.7710(11) 0.033(4) Uani 1 1 d . . . . .
H35 H 1.7116 1.0041 0.7658 0.040 Uiso 1 1 calc R U . . .
C34 C 1.5652(12) 0.9278(10) 0.7072(10) 0.029(3) Uani 1 1 d . . . . .
H34 H 1.5625 0.9036 0.6577 0.035 Uiso 1 1 calc R U . . .
C37 C 0.9356(11) 0.6181(10) 0.0619(9) 0.026(3) Uani 1 1 d . . . . .
H37 H 0.8884 0.5938 0.0005 0.031 Uiso 1 1 calc R U . . .
C38 C 1.0092(11) 0.6115(10) 0.0480(9) 0.027(3) Uani 1 1 d . . . . .
H38 H 1.0181 0.5620 0.0522 0.033 Uiso 1 1 calc R U . . .
C36 C 0.8696(12) 0.5463(12) 0.0805(10) 0.036(4) Uani 1 1 d . . . . .
H36A H 0.8430 0.4932 0.0382 0.043 Uiso 1 1 calc R U . . .
H36B H 0.8139 0.5564 0.0755 0.043 Uiso 1 1 calc R U . . .
C39 C 1.2050(11) 0.6908(9) 0.2492(9) 0.023(3) Uani 1 1 d . . . . .
H39A H 1.2011 0.6545 0.2030 0.027 Uiso 1 1 calc R U . . .

H39B H 1.1379 0.6825 0.2339 0.027 Uiso 1 1 calc R U . . .
C41 C 1.2632(12) 0.8484(9) 0.3153(9) 0.027(3) Uani 1 1 d . . . . .
H41 H 1.2459 0.8063 0.3465 0.033 Uiso 1 1 calc R U . . .
C40 C 1.2702(13) 0.7856(10) 0.2595(10) 0.031(4) Uani 1 1 d . . . . .
H40A H 1.3391 0.7956 0.2838 0.037 Uiso 1 1 calc R U . . .
H40B H 1.2504 0.7962 0.2034 0.037 Uiso 1 1 calc R U . . .
C42 C 1.1772(14) 0.8546(10) 0.2923(11) 0.034(4) Uani 1 1 d . . . . .
H42 H 1.1193 0.8166 0.2430 0.041 Uiso 1 1 calc R U . . .
C45 C 1.4856(10) 0.9396(9) 0.7839(9) 0.022(3) Uani 1 1 d . . . . .
H45 H 1.4280 0.9234 0.7889 0.026 Uiso 1 1 calc R U . . .
C43 C 1.6582(14) 1.0252(10) 0.8423(11) 0.038(4) Uani 1 1 d . . . . .
H43 H 1.7186 1.0670 0.8869 0.046 Uiso 1 1 calc R U . . .
C44 C 1.5742(14) 1.0009(11) 0.8479(11) 0.038(4) Uani 1 1 d . . . . .
H44 H 1.5766 1.0267 0.8966 0.046 Uiso 1 1 calc R U . . .
C46 C 1.0685(14) 0.6748(13) 0.0285(11) 0.044(5) Uani 1 1 d . . . . .
H46 H 1.1158 0.6678 0.0172 0.052 Uiso 1 1 calc R U . . .
C47 C 1.0586(13) 0.7481(12) 0.0255(12) 0.043(5) Uani 1 1 d . . . . .
H47 H 1.1018 0.7931 0.0150 0.052 Uiso 1 1 calc R U . . .
C49 C 0.9277(15) 0.6925(13) 0.0579(12) 0.043(5) Uani 1 1 d . . . . .
H49 H 0.8808 0.7001 0.0694 0.052 Uiso 1 1 calc R U . . .
C48 C 0.9868(17) 0.7568(13) 0.0376(13) 0.050(5) Uani 1 1 d . . . . .
H48 H 0.9777 0.8061 0.0321 0.060 Uiso 1 1 calc R U . . .
C51 C 0.9721(12) 0.0724(9) 0.4606(10) 0.030(3) Uani 1 1 d . . . . .
H51 H 0.9647 0.1233 0.4455 0.035 Uiso 1 1 calc R U . . .
C55 C 0.8759(13) 0.0349(10) 0.4461(10) 0.033(4) Uani 1 1 d . . . . .
H55 H 0.8619 0.0447 0.4902 0.039 Uiso 1 1 calc R U . . .
C50 C 1.0535(14) 0.1258(10) 0.5469(11) 0.041(5) Uani 1 1 d . . . . .
H50A H 1.1164 0.1330 0.5507 0.049 Uiso 1 1 calc R U . . .
H50B H 1.0442 0.0956 0.5886 0.049 Uiso 1 1 calc R U . . .
C52 C 0.9916(17) 0.0593(11) 0.3951(14) 0.046(5) Uani 1 1 d . . . . .
H52 H 1.0573 0.0839 0.4052 0.055 Uiso 1 1 calc R U . . .
C54 C 0.820(2) -0.0282(13) 0.3027(13) 0.060(7) Uani 1 1 d . . . . .
H54 H 0.7675 -0.0628 0.2490 0.072 Uiso 1 1 calc R U . . .
C53 C 0.916(2) 0.0099(14) 0.3139(13) 0.064(8) Uani 1 1 d . . . . .
H53 H 0.9283 0.0027 0.2686 0.076 Uiso 1 1 calc R U . . .
C59 C 1.3474(14) 0.9021(10) 0.3874(11) 0.041(5) Uani 1 1 d . . . . .
H59 H 1.4068 0.8983 0.4029 0.049 Uiso 1 1 calc R U . . .
C58 C 1.3436(16) 0.9624(11) 0.4374(11) 0.044(5) Uani 1 1 d . . . . .
H58 H 1.4001 0.9976 0.4890 0.053 Uiso 1 1 calc R U . . .
C56 C 1.1753(16) 0.9159(13) 0.3407(14) 0.049(5) Uani 1 1 d . . . . .
H56 H 1.1161 0.9201 0.3245 0.058 Uiso 1 1 calc R U . . .
C57 C 1.260(2) 0.9718(12) 0.4133(15) 0.057(6) Uani 1 1 d . . . . .
H57 H 1.2592 1.0157 0.4455 0.068 Uiso 1 1 calc R U . . .
C62 C 0.3800(12) 0.1989(11) 0.1471(11) 0.033(4) Uani 1 1 d . . . . .
H62 H 0.3975 0.1544 0.1438 0.040 Uiso 1 1 calc R U . . .
C61 C 0.2809(12) 0.1814(13) 0.1158(11) 0.038(4) Uani 1 1 d . . . . .

H61 H 0.2319 0.1246 0.0919 0.045 Uiso 1 1 calc R U . . .
C60 C 0.2542(13) 0.2452(15) 0.1194(13) 0.050(5) Uani 1 1 d . . . . .
H60 H 0.1874 0.2328 0.0985 0.061 Uiso 1 1 calc R U . . .
C65 C 0.6902(14) 0.1330(12) 0.0586(10) 0.038(4) Uani 1 1 d . . . . .
H65 H 0.6613 0.0944 0.0022 0.046 Uiso 1 1 calc R U . . .
C63 C 0.8395(12) 0.1413(12) 0.1820(10) 0.035(4) Uani 1 1 d . . . . .
H63A H 0.8794 0.1966 0.1779 0.042 Uiso 1 1 calc R U . . .
H63B H 0.8808 0.1116 0.2008 0.042 Uiso 1 1 calc R U . . .
C64 C 0.7554(13) 0.0916(11) 0.0982(10) 0.037(4) Uani 1 1 d . . . . .
H64A H 0.7155 0.0365 0.1029 0.044 Uiso 1 1 calc R U . . .
H64B H 0.7803 0.0804 0.0604 0.044 Uiso 1 1 calc R U . . .
C69 C 0.7234(16) 0.2025(14) 0.0298(11) 0.047(5) Uani 1 1 d . . . . .
H69 H 0.7854 0.2214 0.0323 0.056 Uiso 1 1 calc R U . . .
C67 C 0.5443(14) 0.1453(17) 0.0195(11) 0.056(6) Uani 1 1 d . . . . .
H67 H 0.4812 0.1252 0.0151 0.067 Uiso 1 1 calc R U . . .
C66 C 0.5997(14) 0.1050(13) 0.0530(11) 0.046(5) Uani 1 1 d . . . . .
H66 H 0.5758 0.0575 0.0724 0.055 Uiso 1 1 calc R U . . .
C68 C 0.577(2) 0.214(2) -0.0082(13) 0.074(9) Uani 1 1 d . . . . .
H68 H 0.5369 0.2413 -0.0315 0.089 Uiso 1 1 calc R U . . .
C70 C 0.667(2) 0.2449(18) -0.0028(13) 0.069(8) Uani 1 1 d . . . . .
H70 H 0.6910 0.2937 -0.0206 0.083 Uiso 1 1 calc R U . . .
C71 C 0.2846(15) 0.1755(13) 0.3276(15) 0.054(6) Uani 1 1 d . . . . .
H71 H 0.2619 0.1252 0.2875 0.065 Uiso 1 1 calc R U . . .
C72 C 0.8009(14) -0.0164(11) 0.3679(14) 0.046(5) Uani 1 1 d . . . . .
H72 H 0.7358 -0.0437 0.3589 0.055 Uiso 1 1 calc R U . . .
F5 F 0.0548(19) 1.0633(13) 0.9488(18) 0.149(9) Uani 1 1 d . . . . .
F6 F 0.115(2) 1.0010(18) 0.891(2) 0.195(14) Uani 1 1 d . . . . .
P3 P 0.0193(11) 0.9701(8) 0.9007(9) 0.144(5) Uani 1 1 d . . . . .
F7 F 0.002(2) 0.8886(14) 0.8665(18) 0.142(9) Uani 1 1 d . . . . .
F8 F -0.063(3) 0.935(2) 0.916(3) 0.29(3) Uani 1 1 d . . . . .
F11 F -0.0429(15) 1.0101(17) 0.8186(11) 0.147(10) Uani 1 1 d . . . . .
F13 F 0.093(4) 0.955(2) 0.988(3) 0.247(19) Uani 1 1 d . . . . .

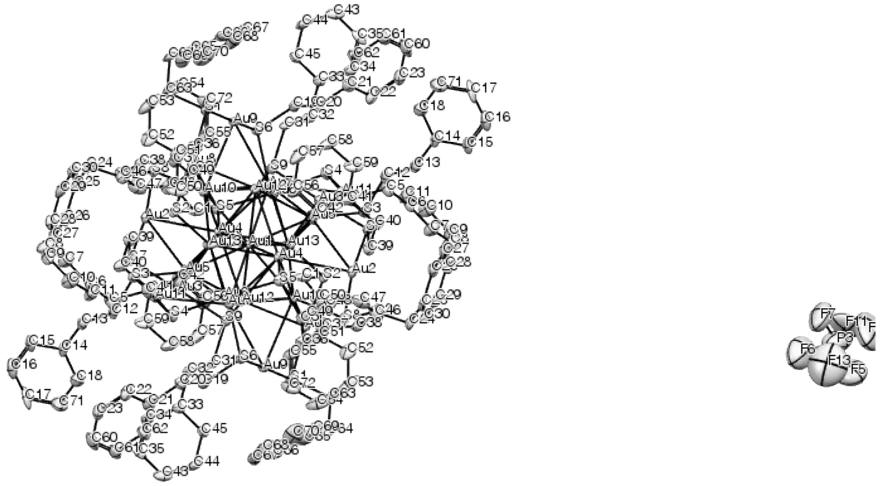

[1] Parker, J. F., Weaver, J. E. F., McCallum, F., Fields-Zinna, C. A. & Murray, R. W *Langmuir* **26,** 13650–13654 (2010)
[2] Sheldrick, G. M. *SADABS – a program for area detector absorption corrections*.
[3] Sheldrick, G, M. *SHELXTL*, v. 6.12; Bruker AXS: Madison, WI, 1999.
[4] Panini, P., Chopra, D. *CrystEngComm* **15**, 3711-3733 (2013
